# Epidemiological transitions of Visceral Leishmaniasis in G20 countries: A 33-year retrospective joinpoint and demographic decomposition analysis with 20-year ARIMA projections

**DOI:** 10.1371/journal.pntd.0014714

**Published:** 2026-09-16

**Authors:** Yongbin Wang, Renfang Zhang, Zhenzhou Liu, Chunjie Xu, Fei Lin

**Affiliations:** 1 Department of Epidemiology and Health Statistics, School of Public Health, Henan Medical University, Xinxiang, Henan, People’s Republic of China; 2 Beijing Key Laboratory of Antimicrobial Agents/Laboratory of Pharmacology, Institute of Medicinal Biotechnology, Chinese Academy of Medical Sciences & Peking Union Medical College, Beijing, China; University of Lucknow, INDIA

## Abstract

**Background:**

Visceral leishmaniasis (VL) remains a fatal neglected tropical disease with substantial global burden. The Group of Twenty (G20) encompasses both endemic and non-endemic nations, yet comprehensive multi-country assessments of VL trends and future projections are limited. This study aims to evaluate the 33-year epidemiological transition of VL across G20 countries and forecast its trajectory to 2043.

**Methodology/Principal findings:**

We extracted VL-related prevalence, incidence, mortality, and disability-adjusted life years (DALYs) data for G20 countries from the Global Burden of Disease 2023 database (1990–2023). We employed joinpoint regression to identify trend inflection points, decomposition analysis to quantify drivers of change (population growth, aging, and epidemiological changes), and autoregressive integrated moving average (ARIMA) models to project trends to 2043. From 1990 to 2023, G20 countries showed significant declines in age-standardized prevalence rate (EAPC = -9.06%), incidence rate (EAPC = -9.06%), mortality rate (EAPC = -7.23%), and DALYs rate (EAPC = -7.40%). Saudi Arabia, Turkey, and India experienced the steepest declines, while Brazil exhibited the smallest reduction and the highest burden in 2023 (age-standardized DALY rate: 30.54 per 100,000). Burden was consistently higher in males and concentrated in children under 5 years, with a slight increase observed among those aged ≥95 years. Decomposition analysis revealed that changes in epidemiological rates were the primary driver of mortality (contributing -82.05%) and DALYs (-52.62%) reductions, counteracting the positive effects of population growth and aging. ARIMA projections (2024–2043) suggest a stabilization in mortality, a gradual decline in DALYs, and a subtle increase in incidence and prevalence rates, with persistent gender disparities.

**Conclusions/Significance:**

While the VL burden in G20 nations has significantly decreased, substantial national disparities are evident. Our analysis specifically identifies Brazil as the priority target for intensified control. Sustained surveillance and international cooperation are crucial to prevent a projected mild resurgence in new infections.

## Author summary

VL is a deadly parasitic disease that disproportionately affects vulnerable populations in tropical and subtropical regions. The G20 brings together the world’s largest economies, including countries where VL is common (such as India and Brazil) and those where it is rare (such as the United States and Germany). In this study, we analyzed VL trends across G20 countries from 1990 to 2023 and projected future patterns through 2043. We found that VL burden has dropped dramatically over the past three decades, largely due to successful vector control, better diagnostics, and improved treatments. However, progress has been uneven—Brazil now carries the heaviest burden among G20 nations, while India, Turkey, and Saudi Arabia achieved the greatest reductions. Young children and males remain most affected, and we observed a concerning slight rise in VL among the very elderly. Looking ahead, we project a mild rebound in new infections, highlighting the need for sustained vigilance. Our findings provide an evidence base for G20 policymakers to allocate resources effectively, strengthen cross-border cooperation, and address emerging challenges such as climate change and population mobility that may reshape VL transmission dynamics in the coming decades.

## Introduction

Visceral Leishmaniasis (VL), also known as kala-azar, is a life-threatening neglected tropical disease (NTD) caused by the Leishmania donovani complex [1]. It is primarily transmitted through the bites of infected female sandflies belonging to the genera Phlebotomus and Lutzomyia [1]. Characterized by core symptoms including prolonged fever, splenomegaly, hepatomegaly, anemia and weight loss, VL is one of the most lethal parasitic diseases globally, with a mortality rate exceeding 90% in untreated patients [2]. According to the World Health Organization (WHO), there are approximately 500,000 new VL cases worldwide each year, over 90% of which are concentrated in three major endemic regions: South Asia (India, Bangladesh, Nepal), East Africa (Sudan, Ethiopia, Kenya) and Latin America (Brazil) [3]. Beyond direct clinical harm, VL imposes substantial socioeconomic burdens on affected populations, including high medical expenses, loss of labor productivity and long-term disability, further exacerbating health inequities in resource-scarce areas [4].

The Group of Twenty (G20) consists of 19 major economies plus the European Union. Collectively, its member states account for around 85% of the global gross domestic product (GDP), 75% of international trade and 60% of the world’s population [5]. Notably, the G20 includes both VL-endemic countries (e.g., India, Brazil, South Africa) and non-endemic high-income countries (e.g., the United States, Germany, Japan) [6]. With the growing frequency of global travel, immigration and cross-border population mobility, these non-endemic countries face potential risks of imported VL cases [7]. G20 members exhibit marked disparities in VL epidemiological profiles, ranging from sustained transmission in hyperendemic areas to sporadic occurrence of local or imported cases in low-endemic regions, posing unique challenges to VL prevention and control efforts [8]. Therefore, The G20’s unique composition makes it a critical unit of analysis for understanding VL dynamics, importation risks, and the differential impact of control policies.

Over the past three decades, global VL control initiatives have primarily focused on vector control, early diagnosis and effective treatment, but progress varies considerably across countries [9]. Although previous studies have assessed VL burden in individual G20 countries or specific regions, a comprehensive cross-country analysis of 1990–2023 trends is lacking [6,10]. Additionally, systematic long-term projections for 2024–2043 are critical for guiding resource allocation and strategy formulation, and research on such projections is insufficient [11].

Building upon the existing research background and identified gaps, this study aims to: (1) retrospectively analyze the trends in the burden of VL—including incidence, prevalence, Disability-adjusted life years (DALYs), and mortality—in G20 countries from 1990 to 2023 using the joinpoint regression; (2) project the future trajectory of VL burden in these countries from 2024 to 2043 using the Autoregressive Integrated Moving Average (ARIMA) model; and (3) identify the key factors that influence both the historical trends and future outcomes of VL burden in G20 nations using the demographic decomposition analysis. The findings of this study are intended to fill the gap in the assessment and prediction of VL burden in G20 countries, enrich relevant evidence, provide an evidence-based basis for member states to formulate prevention and control strategies, allocate resources and strengthen cooperation, and thus contribute to the advancement of the global goal of VL elimination.

## Materials and methods

### Ethics statement

The institutional review board of Henan Medical University approved this study protocol (No: XYLL-2019072), and it is not required to obtain ethical approval because all data in this study were obtained using the GBD outcome tool available on the IHME website (http://ghdx.healthdata.org/).

### Study design and data source

This ecological trend study conducted a secondary analysis of publicly available, aggregate data from the GBD 2023 study (http://ghdx.healthdata.org/). The GBD 2023 study provides standardized, comparative estimates of disease burden for 375 diseases and injuries and 88 risk factors across 204 countries and territories, including 660 subnational locations, from 1990 to 2023 [8,12]. The GBD study uses an integrated approach, sourcing data from vital registration systems, censuses, and published studies to ensure the robustness and representativeness of the dataset [13]. We extracted data for all G20 member states on VL-related prevalence, incidence, mortality, and DALYs; however, the GBD 2023 database provided complete and reliable VL burden estimates for only 10 of the 19 G20 member states (Argentina, Brazil, China, France, India, Italy, Mexico, Saudi Arabia, Turkey, and the European Union). Data for the remaining nine countries and Russia were unavailable or incomplete and were therefore excluded from the analysis.

### Statistical analysis

We extracted counts and rates (per 100,000 population) of VL prevalence, incidence, mortality, and DALYs. Age-standardized prevalence rates (ASPR), age-standardized incidence rates (ASIR), age-standardized mortality rates (ASMR) and age-standardized DALYs rates (ASDR) were calculated using the GBD standard population structure to enable comparison across countries and over time. To quantify temporal trends from 1990 to 2023, we calculated the Estimated Annual Percentage Change (EAPC) and its 95% confidence interval (CI) for each ASR using a log-linear regression model [14].

To identify significant inflection points in temporal trends, we performed joinpoint regression analysis using Joinpoint Regression Software (Version 5.2.0.0, National Cancer Institute). A maximum of 5 joinpoints was allowed, consistent with standard practice for time series of this length (33 years) [15]. This choice balances the need to detect meaningful trend changes against the risk of overfitting, as each segment requires a minimum of 5 years for stable APC estimation. Allowing more joinpoints would produce segments with insufficient data points, while allowing fewer would risk missing clinically important inflection points. The optimal number of joinpoints was selected using a Monte Carlo permutation test (4499 permutations) [15]. For each identified segment, the Annual Percentage Change (APC) was calculated, and the Average Annual Percentage Change (AAPC) was derived to summarize the trend over the entire period.

To disentangle the drivers of changes in the absolute number of VL related burden indicators between 1990 and 2023, we performed a demographic decomposition analysis based on the method by Das Gupta [16]. Decomposition analysis partitions the total change in the number of events (e.g., deaths) between 1990 and 2023 into three components: (1) the effect of total population growth, (2) the effect of changes in the population age structure (aging), and (3) the effect of changes in the age-specific epidemiological rates (e.g., mortality rate). For a given indicator, let $P_{i}\left(t\right)$ be the population size and $R_{i}\left(t\right)$ be the age-specific rate in age group *i* at time *t*. The total change is the sum across age groups of $P_{i}\left(t_{\text{2}}\right)R_{i}\left(t_{\text{2}}\right)-P_{i}\left(t_{\text{1}}\right)R_{i}\left(t_{\text{1}}\right)$. The contribution of the rate effec*t*, for example, is calculated as $\sum_{i}\left[\frac{\left(P_{i}\left(t_{\text{2}})+P_{i}(t_{\text{1}}\right)\right)\times \left(R_{i}\left(t_{\text{2}})-R_{i}(t_{\text{1}}\right)\right)}{\text{2}}\right]$, with analogous symmetric formulations for population growth and aging effects. A key feature of this method is that all interaction effects are distributed proportionally across the three main components, thereby avoiding a separate, uninterpretable interaction term. A positive contribution indicates that a factor increased the number of events, while a negative contribution indicates it decreased the number.

The ARIMA model was used to project trends in ASPR, ASIR, ASMR and ASDR from 2024 to 2043 [17]. The optimal ARIMA(p,d,q) model for each time series was identified using the auto.arima() function in R, which minimizes the Akaike Information Criterion (AIC) [17]. The predictive performance of each model was validated through backtesting on data from 2014 to 2023, with accuracy assessed using the Mean Absolute Percentage Error (MAPE). A MAPE value of < 25% was considered indicative of good predictive performance.

All statistical analyses were conducted using R software (Version 4.5.2, R Foundation for Statistical Computing). A *P*-value < 0.05 was considered statistically significant.

## Results

### Trends in prevalence of VL among G20 countries, 1990–2023

**Fig 1:**
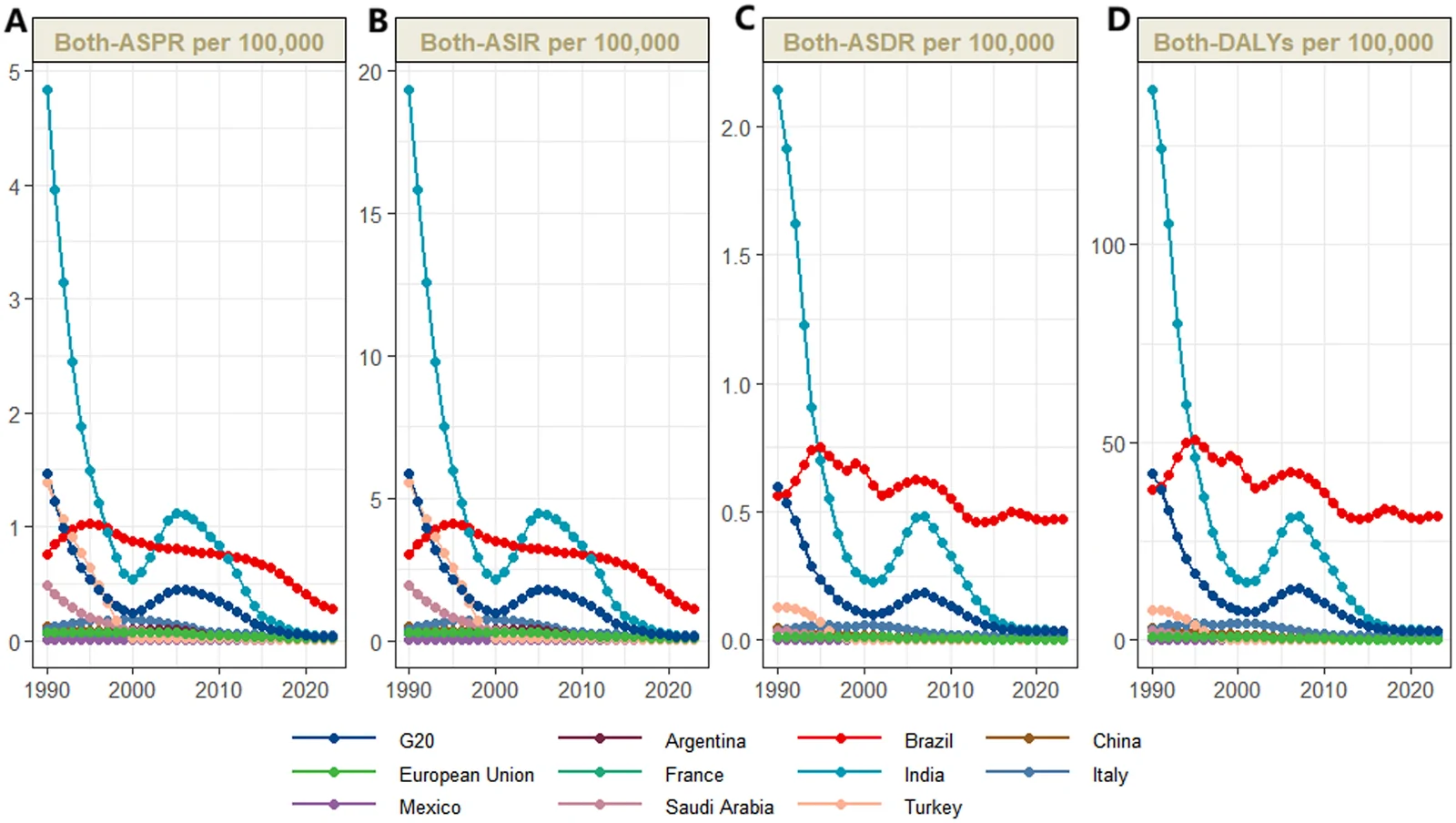
Temporal trends in age-standardized rates of visceral leishmaniasis (VL) across G20 countries, 1990–2023. (A) Prevalence; (B) Incidence; (C) Mortality; (D) Disability-adjusted life years (DALYs). Shaded areas represent 95% uncertainty intervals. The dashed vertical line at 2011 indicates the inflection point marking the onset of a steep decline in most indicators, as identified by joinpoint regression.

**Fig 2:**
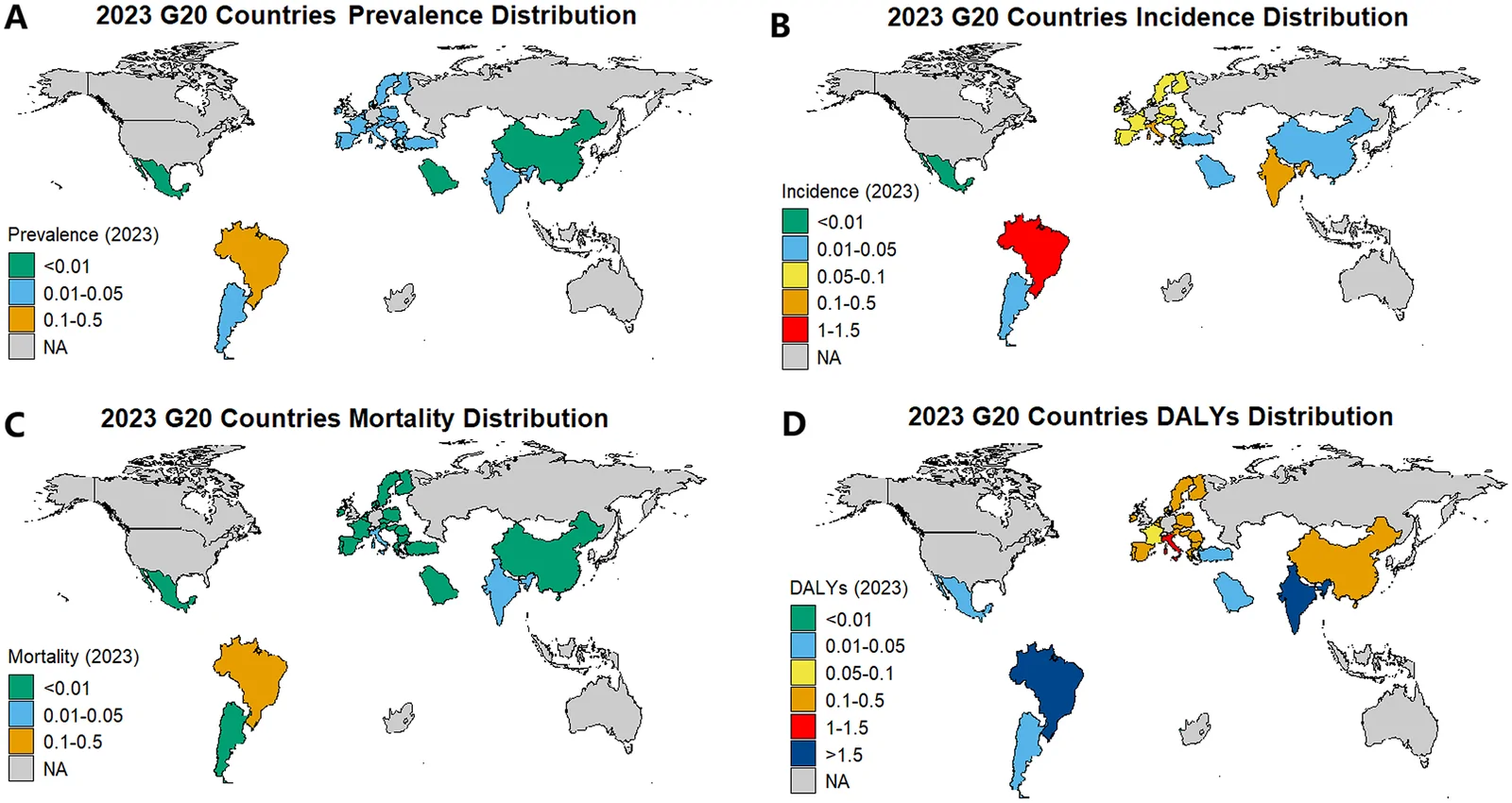
Geographic distribution of visceral leishmaniasis (VL) burden in G20 countries for both sexes, 2023. (A) Age-standardized prevalence rate (ASPR); (B) Age-standardized incidence rate (ASIR); (C) Age-standardized mortality rate (ASMR); (D) Age-standardized DALYs rate (ASDR). Data are displayed per 100,000 population. Countries with no available estimates in the GBD database are shaded in grey. Note the substantially higher burden in Brazil compared to other member states. Base map boundaries were obtained from the public‑domain world dataset in the R ‘maps’ package (version 4.5.2; https://cran.r-project.org/web/packages/maps/index.html), which is derived from the Natural Earth project (https://www.naturalearthdata.com/). Natural Earth data are in the public domain (CC0).

From 1990 to 2023, the ASPR of VL for the overall G20 exhibited a statistically significant decreasing trend (*P* < 0.05), with an EAPC of -9.06% (95% CI: -10.46 to -7.63). In 1990, the number of VL cases was 52,133.45 (95% UI: 31,505.95 to 82,717.14), with an ASPR of 1.46 per 100,000 population (95% UI: 0.88 to 2.32). By 2023, these figures had dropped sharply to 1,373.66 cases (95% UI: 1,203.37 to 1,649.46) and an ASPR of 0.04 per 100,000 population (95% UI: 0.03 to 0.04) (Table 1 and Fig 1A). A decreasing trend of varying magnitudes was also observed across individual G20 member states. The most marked declines were recorded in Saudi Arabia (EAPC = -13.53%, 95% CI: -14.02 to -13.04), Turkey (EAPC = -13.40%, 95% CI: -15.96 to -10.76), and India (EAPC = -11.38%, 95% CI: -13.12 to -9.60). In contrast, Brazil exhibited a relatively moderate reduction (EAPC = -2.82%, 95% CI: -3.43 to -2.20) but had the highest number (621.57 cases, 95% UI: 511.80 to 757.75) and ASPR (0.35 per 100,000 population, 95% UI: 0.29 to 0.42) of VL cases among G20 countries in 2023. Statistically significant decreases (all *P* < 0.05) were additionally noted in Argentina (EAPC = -7.78%), China (EAPC = -7.62%), the European Union (EAPC = -4.53%), France (EAPC = -4.58%), Italy (EAPC = -5.18%), and Mexico (EAPC = -3.31%) (Table 1 and Figs 1A, 2A and S1A).

**Table 1:** Burden of visceral leishmaniasis (VL) in G20 Countries, 1990 and 2023: prevalent and incident cases, deaths, and disability-adjusted life years (DALYs) with age-standardized rates and estimated annual percentage change (EAPC).

Index	Prevalence (95% UI)				
	1990		2023		1990-2023
	Prevalent cases	ASPR per 100,000	Prevalent cases	ASPR per 100,000	EAPC (95%CI), %
G20	52133.45 (31505.95-82717.14)	1.46 (0.88-2.32)	1373.66 (1203.37-1649.46)	0.04 (0.03-0.04)	-9.06 (-10.46 to -7.63)
Argentina	22.34 (7.95-50)	0.07 (0.02-0.15)	4.26 (2.46-6.76)	0.01 (0.01-0.02)	-7.78 (-9.32 to -6.22)
Brazil	1268.70 (896.14-1704.28)	0.75 (0.53-1.01)	621.57 (511.80-757.75)	0.35 (0.29-0.42)	-2.82 (-3.43 to -2.2)
China	1378.09 (541.95-2992.18)	0.12 (0.05-0.26)	121.51 (53.59-253.46)	0.01 (0.00-0.02)	-7.62 (-8.14 to -7.11)
European Union	257.34 (128.19-457.28)	0.07 (0.04-0.13)	62.75 (41.75-89.14)	0.02 (0.01-0.03)	-4.53 (-5.14 to -3.92)
France	27.14 (10.68-56.44)	0.05 (0.02-0.11)	8.05 (4.95-12.07)	0.02 (0.01-0.02)	-4.58 (-5.44 to -3.72)
India	48389.53 (29323.49-76958.32)	4.83 (2.92-7.72)	589.04 (384.24-925.08)	0.04 (0.03-0.07)	-11.38 (-13.12 to -9.6)
Italy	43.74 (28.63-63.64)	0.10 (0.07-0.15)	15.92 (9.29-23.99)	0.04 (0.02-0.06)	-5.18 (-6.07 to -4.28)
Mexico	4.55 (2.36-7.68)	0.00 (0.00-0.01)	2.04 (1.18-3.41)	0.00 (0.00-0.00)	-3.31 (-4.03 to -2.59)
Saudi Arabia	98.30 (38.31-203.56)	0.48 (0.19-0.99)	1.82 (0.76-3.96)	0.01 (0.00-0.01)	-13.53 (-14.02 to -13.04)
Turkey	901.04 (424.80-1646.35)	1.39 (0.65-2.55)	9.44 (4.07-19.70)	0.01 (0.01-0.03)	-13.4 (-15.96 to -10.76)
**Index**	**Incidence (95% UI)**				
	**1990**		**2023**		**1990-2023**
	**Incident cases**	**ASIR per 100,000**	**Incident cases**	**ASIR per 100,000**	**EAPC (95%CI), %**
G20	208533.80 (126023.82-330868.59)	5.84 (3.53-9.27)	5495.25 (4813.96-6598.67)	0.14 (0.12-0.17)	-9.06 (-10.46 to -7.63)
Argentina	89.38 (31.81-200)	0.26 (0.09-0.59)	17.04 (9.83-27.03)	0.04 (0.02-0.07)	-7.78 (-9.32 to -6.22)
Brazil	5074.80 (3584.57-6817.14)	3.01 (2.12-4.04)	2486.41 (2047.28-3031.16)	1.39 (1.14-1.69)	-2.82 (-3.43 to -2.20)
China	5512.38 (2167.82-11968.71)	0.47 (0.19-1.02)	486.05 (214.37-1013.86)	0.04 (0.02-0.09)	-7.62 (-8.14 to -7.11)
European Union	1029.36 (512.76-1829.12)	0.30 (0.15-0.53)	250.99 (166.98-356.56)	0.07 (0.05-0.11)	-4.53 (-5.14 to -3.93)
France	108.56 (42.72-225.76)	0.22 (0.09-0.46)	32.22 (19.82-48.28)	0.06 (0.04-0.09)	-4.58 (-5.44 to -3.72)
India	193558.13 (117293.98-307833.27)	19.32 (11.66-30.89)	2356.68 (1537.28-3701.08)	0.17 (0.11-0.27)	-11.38 (-13.12 to -9.60)
Italy	174.96 (114.51-254.54)	0.42 (0.27-0.61)	63.67 (37.16-95.96)	0.16 (0.09-0.24)	-5.18 (-6.07 to -4.28)
Mexico	18.22 (9.44-30.74)	0.02 (0.01-0.03)	8.16 (4.70-13.63)	0.01 (0.00-0.01)	-3.31 (-4.03 to -2.59)
Saudi Arabia	393.20 (153.23-814.24)	1.91 (0.75-3.96)	7.27 (3.02-15.86)	0.02 (0.01-0.05)	-13.53 (-14.02 to -13.04)
Turkey	3604.18 (1699.21-6585.38)	5.56 (2.62-10.19)	37.74 (16.28-78.78)	0.05 (0.02-0.11)	-13.40 (-15.96 to -10.76)
**Types**	**Deaths (95% UI)**				
	**1990**		**2023**		**1990-2023**
	**Deaths**	**ASMR per 100,000**	**Deaths**	**ASMR per 100,000**	**EAPC (95%CI), %**
G20	20449.28 (20.66-65178.01)	0.60 (0.00-1.90)	1538.22 (8.88-4570.75)	0.04 (0.00-0.11)	-7.23 (-8.28 to -6.17)
Argentina	0.54 (0.00-1.59)	0.00 (0.00-0.00)	0.16 (0.00-0.47)	0.00 (0.00-0.00)	-3.88 (-4.65 to -3.09)
Brazil	865.13 (0.35-2644.59)	0.56 (0.00-1.69)	910.32 (0.44-2638.01)	0.46 (0.00-1.35)	-1.29 (-1.59 to -0.99)
China	517.76 (0.13-1667.87)	0.05 (0.00-0.15)	72.15 (0.02-230.55)	0.01 (0.00-0.02)	-6.10 (-6.47 to -5.73)
European Union	53.92 (12.47-132.47)	0.01 (0.00-0.03)	14.72 (6.43-29.21)	0.00 (0.00-0.01)	-4.75 (-5.18 to -4.32)
France	2.88 (0.00-8.58)	0.00 (0.00-0.01)	0.63 (0.00-1.90)	0.00 (0.00-0.00)	-5.74 (-6.15 to -5.33)
India	18969.59 (5.71-60706.91)	2.14 (0.00-6.85)	545.60 (0.15-1717.50)	0.04 (0.00-0.13)	-10.41 (-11.84 to -8.95)
Italy	14.98 (11.34-18.49)	0.04 (0.03-0.04)	8.04 (6.11-9.91)	0.02 (0.01-0.02)	-4.23 (-5.04 to -3.42)
Mexico	0.90 (0.00-2.75)	0.00 (0.00-0.00)	0.45 (0.00-1.37)	0.00 (0.00-0.00)	-3.41 (-4.21 to -2.61)
Saudi Arabia	6.60 (0.00-21.34)	0.04 (0.00-0.13)	0.13 (0.00-0.40)	0.00 (0.00-0.00)	-13.38 (-13.77 to -12.98)
Turkey	70.89 (0.02-225.85)	0.13 (0.00-0.42)	0.74 (0.00-2.23)	0.00 (0.00-0.00)	-13.81 (-16.47 to -11.06)
**Types**	**DALYs (95% UI)**				
	**1990**		**2023**		**1990-2023**
	**DALYs**	**ASDR per 100,000**	**DALYs**	**ASDR per 100,000**	**EAPC (95%CI), %**
G20	1471937.54 (4986.10-4689536.41)	42.02 (0.14-134)	95146.94 (618.57-284175.62)	2.46 (0.02-7.35)	-7.40 (-8.46 to -6.33)
Argentina	33.90 (0.80-97.16)	0.10 (0.00-0.29)	8.75 (0.22-25.30)	0.02 (0.00-0.06)	-4.07 (-4.80 to -3.32)
Brazil	62431.72 (120.56-192110.22)	37.92 (0.07-116.21)	55746.07 (63.50-164737.68)	30.54 (0.04-89.98)	-1.37 (-1.68 to -1.07)
China	35535.39 (72.69-113616.62)	3.10 (0.01-9.94)	3916.48 (8.29-12546.92)	0.34 (0.00-1.10)	-6.23 (-6.61 to -5.85)
European Union	3048.41 (861.43-7140.39)	0.89 (0.26-2.08)	749.66 (381.13-1364.52)	0.23 (0.13-0.40)	-4.95 (-5.38 to -4.52)
France	147.35 (1.23-433.94)	0.29 (0.00-0.86)	27.57 (0.47-81.70)	0.05 (0.00-0.14)	-5.79 (-6.22 to -5.36)
India	1367601.95 (3614.73-4386925.85)	139.01 (0.36-444.65)	34899.86 (54.03-111659.56)	2.64 (0.00-8.52)	-10.50 (-11.94 to -9.03)
Italy	1008.54 (764.10-1241.92)	2.64 (2.01-3.25)	471.96 (356.47-583.46)	1.39 (1.05-1.73)	-4.37 (-5.18 to -3.56)
Mexico	66.25 (0.32-203.19)	0.07 (0.00-0.20)	28.51 (0.13-87.51)	0.03 (0.00-0.08)	-3.28 (-4.06 to -2.49)
Saudi Arabia	491.64 (4.86-1571.42)	2.38 (0.02-7.60)	7.95 (0.09-24.01)	0.03 (0.00-0.08)	-13.72 (-14.12 to -13.32)
Turkey	4620.80 (50.97-14569.92)	7.60 (0.08-24.05)	39.79 (0.46-118.22)	0.05 (0.00-0.16)	-13.82 (-16.44 to -11.12)

Note: UI, Uncertainty interval; ASPR, Age-standardized prevalence rate (per 100,000); ASIR, Age-standardized incidence rate (per 100,000); ASMR, Age-standardized mortality rate (per 100,000); ASDR, Age-standardized DALY rate (per 100,000). Values of “0.00” indicate figures below 0.005 after rounding; they do not denote absolute zero. Data for other G20 member states are not available in the GBD 2023 database. EAPCs with 95% confidence intervals (CI) are presented; all trends were statistically significant at *P* < 0.05.

### Trends in incidence of VL among G20 countries, 1990–2023

From 1990 to 2023, the ASIR of VL across the overall G20 demonstrated a pronounced decreasing trend that paralleled the trajectory of ASPR, with an EAPC of -9.06% (95% CI: -10.46 to -7.63). In 1990, the total number of VL incident cases stood at 208,533.80 (95% UI: 126,023.82 to 330,868.59), corresponding to an ASIR of 5.84 per 100,000 population (95% UI: 3.53 to 9.27). By 2023, these metrics had plummeted to 5,495.25 cases (95% UI: 4,813.96 to 6,598.67) and ASIR of 0.14 per 100,000 population (95% UI: 0.12 to 0.17) (Table 1 and Fig 1B). All G20 member states exhibited statistically significant reductions in ASIR (*P* < 0.05). The steepest declines were documented in Saudi Arabia (EAPC = -13.53%, 95% CI: -14.02 to -13.04), Turkey (EAPC = -13.40%, 95% CI: -15.96 to -10.76), and India (EAPC = -11.38%, 95% CI: -13.12 to -9.60). Conversely, Brazil registered the most modest reduction (EAPC = -2.82%, 95% CI: -3.43 to -2.20) yet ranked first among G20 nations in VL incidence in 2023, with 2,486.41 cases (95% UI: 2,047.28 to 3,031.16) and an ASIR of 1.39 per 100,000 population (95% UI: 1.14 to 1.69). Argentina, China, the European Union, France, Italy, and Mexico displayed EAPCs that matched those for VL prevalence exactly, with values of -7.78%, -7.62%, -4.53%, -4.58%, -5.18%, and -3.31%, respectively (Table 1 and Figs 1B, 2B and S1B).

### Trends in deaths of VL among G20 countries, 1990–2023

From 1990 to 2023, the ASMR of VL across the overall G20 also presented a statistically significant decreasing trend, with an EAPC of -7.23% (95% CI: -8.28 to -6.17). In 1990, the number of VL-related deaths was 20,449.28 (95% UI: 20.66 to 65,178.01), yielding an ASMR of 0.60 per 100,000 population (95% UI: 0.00 to 1.90). By 2023, these figures had fallen to 1,538.22 deaths (95% UI: 8.88 to 4,570.75) and an ASMR of 0.04 per 100,000 population (95% UI: 0.00 to 0.11), with the magnitude of reduction being less pronounced than that of both prevalence and incidence rates (Table 1 and Fig 1C). Among individual G20 member states, the most dramatic reductions in mortality metrics were observed in Turkey (EAPC = -13.81%, 95% CI: -16.47 to -11.06), Saudi Arabia (EAPC = -13.38%, 95% CI: -13.77 to -12.98), and India (EAPC = -10.41%, 95% CI: -11.84 to -8.95). Brazil again exhibited the least substantial decline (EAPC = -1.29%, 95% CI: -1.59 to -0.99) and emerged as the country with the heaviest VL mortality burden across the G20 in 2023, accounting for 910.32 deaths cases (95% UI: 0.44 to 2,638.01) and an ASMR of 0.46 per 100,000 population (95% UI: 0.00 to 0.11). Statistically significant decreases (*P* < 0.05) were further confirmed for China (EAPC = -6.10%), the European Union (EAPC = -4.75%), France (EAPC = -5.74%), Italy (EAPC = -4.23%), Argentina (EAPC = -3.88%), and Mexico (EAPC = -3.41%), in that order (Table 1 and Figs 1C, 2C and S1C).

### Trends in DALYs of VL among G20 countries, 1990–2023

From 1990 to 2023, the ASDR of VL across the overall G20 continued to show a statistically significant reduction, with an EAPC of -7.40% (95%CI: -8.46 to -6.33). In 1990, the total VL-related DALYs amounted to 1,471,937.54 (95% UI: 4,986.10 to 4,689,536.41), corresponding to an ASDR of 42.02 per 100,000 population (95% UI: 0.14 to 134.00). By 2023, this metric had decreased to 95,146.94 (95% UI: 618.57 to 284,175.62) and an ASDR of 2.46 per 100,000 population (95% UI: 0.02 to 7.35), a trend that was highly consistent with that of the mortality rate (Table 1 and Fig 1D). Statistically significant declines in ASDR were observed across all G20 member states (*P* < 0.05). Turkey (EAPC = -13.82%, 95% CI: -16.44 to -11.12), Saudi Arabia (EAPC = -13.72%, 95% CI: -14.12 to -13.32), and India (EAPC = -10.50%, 95% CI: -11.94 to -9.03) ranked as the top three countries with the greatest reduction magnitudes. Brazil again recorded the least pronounced decrease (EAPC = -1.37%, 95% CI: -1.68 to -1.07) and had far higher ASDR than other member states in 2023, with 55,746.07 DALYs (95% UI: 63.50 to 164,737.68) and an ASDR of 30.54 per 100,000 population (95% UI: 0.04 to 89.98). The EAPCs for China, the European Union, France, Italy, Argentina, and Mexico were -6.23%, -4.95%, -5.79%, -4.37%, -4.07%, and -3.28%, respectively (Table 1 and Figs 1D, 2D and S1D).

### Joinpoint regression analysis of temporal trends, 1990–2023

**Fig 3:**
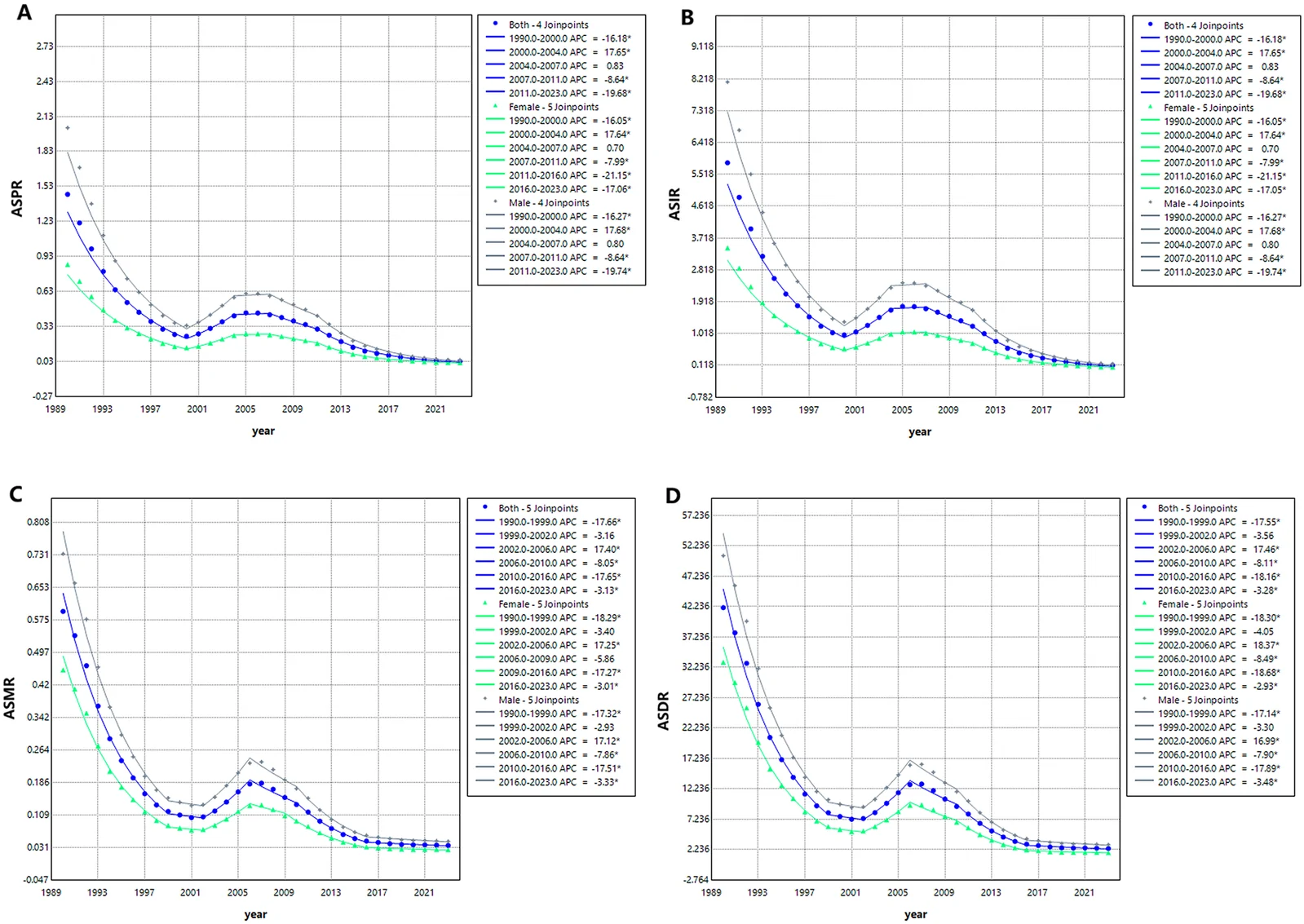
Joinpoint regression analysis of visceral leishmaniasis (VL) age-standardized rates by sex in the G20 aggregate, 1990–2023. (A) Prevalence; (B) Incidence; (C) Mortality; (D) Disability-adjusted life years (DALYs). Solid lines represent fitted trend segments identified by the joinpoint model. The annual percentage change (APC) and its significance (^*^P < 0.05) are displayed for each segment. The distinct trend phases, including a notable decline post-2011, are clearly delineated.

For the overall G20, temporal trends in both-sex ASPR and ASIR exhibited four joinpoints with completely consistent phases: a significant decrease during 1990–2000 (APC = -16.18%, *P* < 0.001), followed by a slight rebound in 2000–2004 (APC = 17.65%, *P* < 0.001), relative stability in 2004–2007 (APC = 0.83%, *P* = 0.79), another decline in 2007–2011 (APC = -8.64%, *P* < 0.001), and a sharp reduction in 2011–2023 (APC = -19.69%, *P* < 0.001). Male ASPR and ASIR trends mirrored those of the both-sex group, showing negligible differences in APC values at corresponding joinpoints. In contrast, five joinpoints were identified in the female group; its first three phases were identical to the both-sex group, succeeded by a substantial decrease in 2011–2016 (APC = -21.15%, *P* < 0.001) and a decelerated reduction in 2016–2023 (APC = -17.06%, *P* < 0.001). Over the entire period from 1990 to 2023, the AAPC in ASPR and ASIR were -11.63% for the both-sex group, -11.69% for males, and -11.17% for females, all demonstrating statistical significance (*P* < 0.001) (Fig 3A and 3B and S1 Table).

Similarly, for the overall G20 both-sex ASMR and ASDR, five joinpoints were detected for each index, revealing similar trend trajectories. These included a marked decrease in 1990–1999 (ASMR: APC = -17.66%, *P* < 0.001; ASDR: APC = -17.55%, *P* < 0.001), a slowed reduction in 1999–2002 (ASMR: APC = -3.16%, *P* = 0.49; ASDR: APC = -3.56%, *P* = 0.45), a rebound in 2002–2006 (ASMR: APC = 17.40%, *P* < 0.001; ASDR: APC = 17.46%, *P* < 0.001), a further decline in 2006–2010 (ASMR: APC = -8.05%, *P* = 0.002; ASDR: APC = -8.11%, *P* = 0.002), a drastic drop in 2010–2016 (ASMR: APC = -17.65%, *P* < 0.001; ASDR: APC = -18.16%, *P* < 0.001), and a moderate decrease in 2016–2023 (ASMR: APC = -3.13%, *P* < 0.001; ASDR: APC = -3.28%, *P* < 0.001). While male and female trends for ASMR and ASDR generally aligned with the both-sex group, with only minor APC variations across certain phases, females consistently exhibited slightly greater reduction magnitudes than males in some periods. From 1990 to 2023, the AAPCs for ASMR and ASDR were -8.49% and -8.62% for the both-sex group, -8.38% and -8.48% for males, and -8.71% and -8.89% for females, respectively, all of which were statistically significant (*P* < 0.001) (Fig 3C and 3D and S1 Table).

From the perspective of high-burden countries (Brazil, India, Italy and Turkey), VL age-standardized indicators showed distinct temporal patterns across 1990–2023. For ASPR and ASIR, India showed five sequential phases: sharp decline, transient rebound, stable period, moderate drop and steep long-term reduction, with a significant AAPC of −14.77%. Turkey had three continuous descending segments and an overall AAPC of −13.96%. Brazil and Italy each had six segmented trends with initial growth followed by persistent declines, with AAPCs of −3.07% and −2.97% (all *P* < 0.001). Regarding ASMR and ASDR, India and Turkey achieved prominent long-term declines with statistically significant negative AAPCs between −12.09% and −14.63%. Italy’s mortality and DALYs burdens also decreased overall (AAPC: −1.85% and −1.99%, *P* < 0.001). In contrast, Brazil’s ASMR and ASDR AAPCs were non-significant (AAPC = −0.52%, *P* = 0.15; AAPC = −0.59%, *P* = 0.100), accompanied by several non-significant short-term oscillations and no obvious long-term downward trend (S2 Fig and S2 Table).

### Age and sex distribution of burden

**Fig 4:**
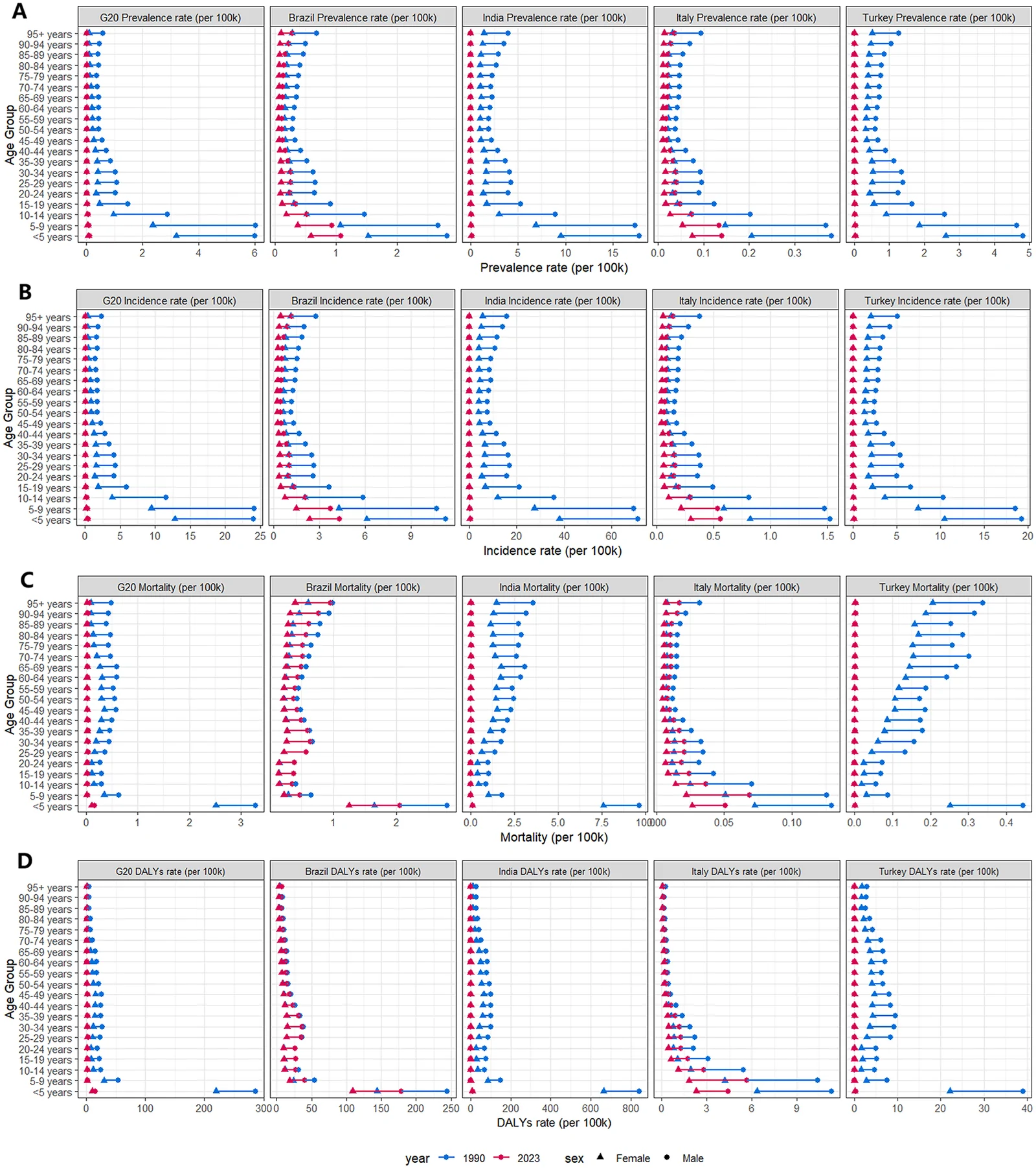
Age- and sex-specific rates of visceral leishmaniasis (VL) in the G20 aggregate and four high-burden countries (Brazil, India, Italy, Turkey) for 1990 and 2023. (A) Prevalence; (B) Incidence; (C) Mortality; (D) Disability-adjusted life years (DALYs). Each panel displays bars for males (M) and females (F) across all age groups. The concentration of burden in children under 5 years and the persistent male predominance are consistently observed. The y-axis scales differ by country to highlight relative within-country age distributions.

In both 1990 and 2023, the ASPR, ASIR, ASMR and ASDR of VL across all age groups in the overall G20 were significantly higher in males than in females. This sex disparity was consistently observed in all high-burden countries (Brazil, India, Italy, and Turkey) and proved statistically significant. In terms of age distribution, the disease burden was predominantly concentrated in children under 5 years of age. In 1990, among males under 5 in the overall G20, the ASPR reached 6.02 per 100,000 population, ASIR 24.07 per 100,000, ASMR 3.28 per 100,000, and ASDR 286.16 per 100,000, all representing the highest levels across all age groups. By 2023, the burden had declined substantially across all age groups, yet children under 5 still remained the most affected demographic, with male ASPR 0.12 per 100,000, ASIR 0.47 per 100,000, ASMR 0.17 per 100,000, and ASDR 14.86 per 100,000. Among high-burden countries, Brazil had markedly higher VL burden indicators across all age groups compared with other nations, and its age distribution characteristics were consistent with those of the overall G20. In India, the disease burden in children under 5 was far higher than that in other countries in 1990; despite a substantial reduction by 2023, young children still constituted the primary affected population. Italy and Turkey exhibited relatively low burden levels across all age groups, with less variations in age distribution. Additionally, although the burden indicators among adults aged 95 years and over were lower than those in the pediatric population, this group showed a slight upward trend in 2023 relative to other middle-aged and elderly cohorts (Fig 4A–4D and S3 Table).

### Decomposition analysis of VL

**Fig 5:**
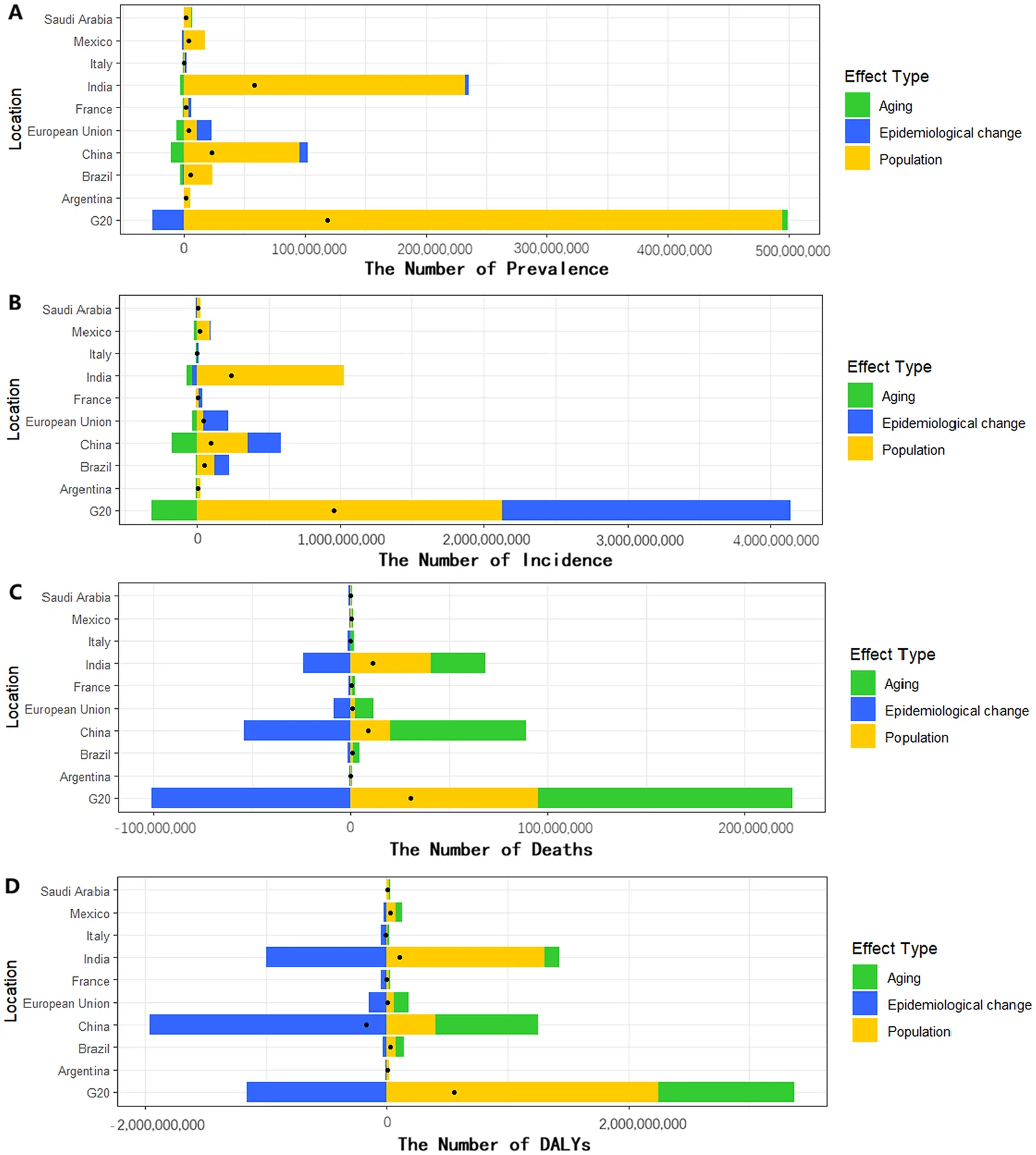
Decomposition analysis of changes in visceral leishmaniasis (VL) burden in G20 countries from 1990 to 2023. Contribution of population growth, population aging, and epidemiological rate changes to the total change in (A) Prevalence cases, (B) Incident cases, (C) Deaths, and (D) Disability-adjusted life years (DALYs). Black dots denote the net total change. Positive bars indicate a factor’s contribution to an increase in burden, while negative bars indicate a contribution to a decrease. This analysis identifies the primary drivers of the epidemiological transition.

From 1990 to 2023, the changes in VL prevalence, incidence, deaths, and DALYs across G20 countries were jointly driven by three factors: population growth, population aging, and variations in epidemiological rates. Notably, the contribution of each factor varied significantly across different indicators and countries. For the overall G20, the change in VL prevalence cases was primarily driven by population growth (contribution: 104.61%), with population aging accounting for a marginal positive contribution (0.90%) and changes in epidemiological rates exerting a negative effect (-5.51%). Population aging had a negative contribution to the change in prevalence cases in Brazil, China, and the European Union, meaning that the aging population structure in these countries decreased the expected number of prevalent cases, likely because VL is more common in younger age groups. By contrast, in Italy, the shift in epidemiological rates (contribution: 113.67%) served as the dominant driver of prevalence case changes, with a positive impact. The variation in VL incidence cases across the overall G20 was positively driven by both population growth (55.83%) and epidemiological rate changes (52.45%), whereas population aging had a negative contribution (-8.28%). In Italy and the European Union, epidemiological rate changes contributed 126.37% and 91.49%, respectively, which were the primary causes of increased incidence cases. In India, however, the impact of epidemiological rate changes on incidence was slightly negative (-3.04%) (Fig 5A and 5B and S4 and S5 Tables).

Regarding the overall G20 mortality changes, population aging (104.96%) and population growth (77.09%) were the major positive contributors, while epidemiological rate changes exerted a pronounced negative effect (-82.05%)—a pattern consistent across most member states. Population aging emerged as the top driver of mortality changes in China (196.56%), Italy (259.66%), and the European Union (279.95%). In Saudi Arabia, where VL mortality showed an overall downward trend, epidemiological rate changes had a strongly negative contribution (-380.05%). Changes in VL DALYs across the overall G20 were positively driven by population aging (50.86%) and population growth (101.76%), with epidemiological rate changes making a negative contribution (-52.62%). In Argentina, China, the European Union, France, and Italy, epidemiological rate changes exerted a markedly negative impact, with contributions as high as -540.18% in the European Union and -276.94% in China. Conversely, the negative contribution of epidemiological rate changes to DALY variations was negligible in Saudi Arabia (-2.95%) (Fig 5C and 5D and S6 and S7 Tables).

### Projected trends, 2024–2043

**Fig 6:**
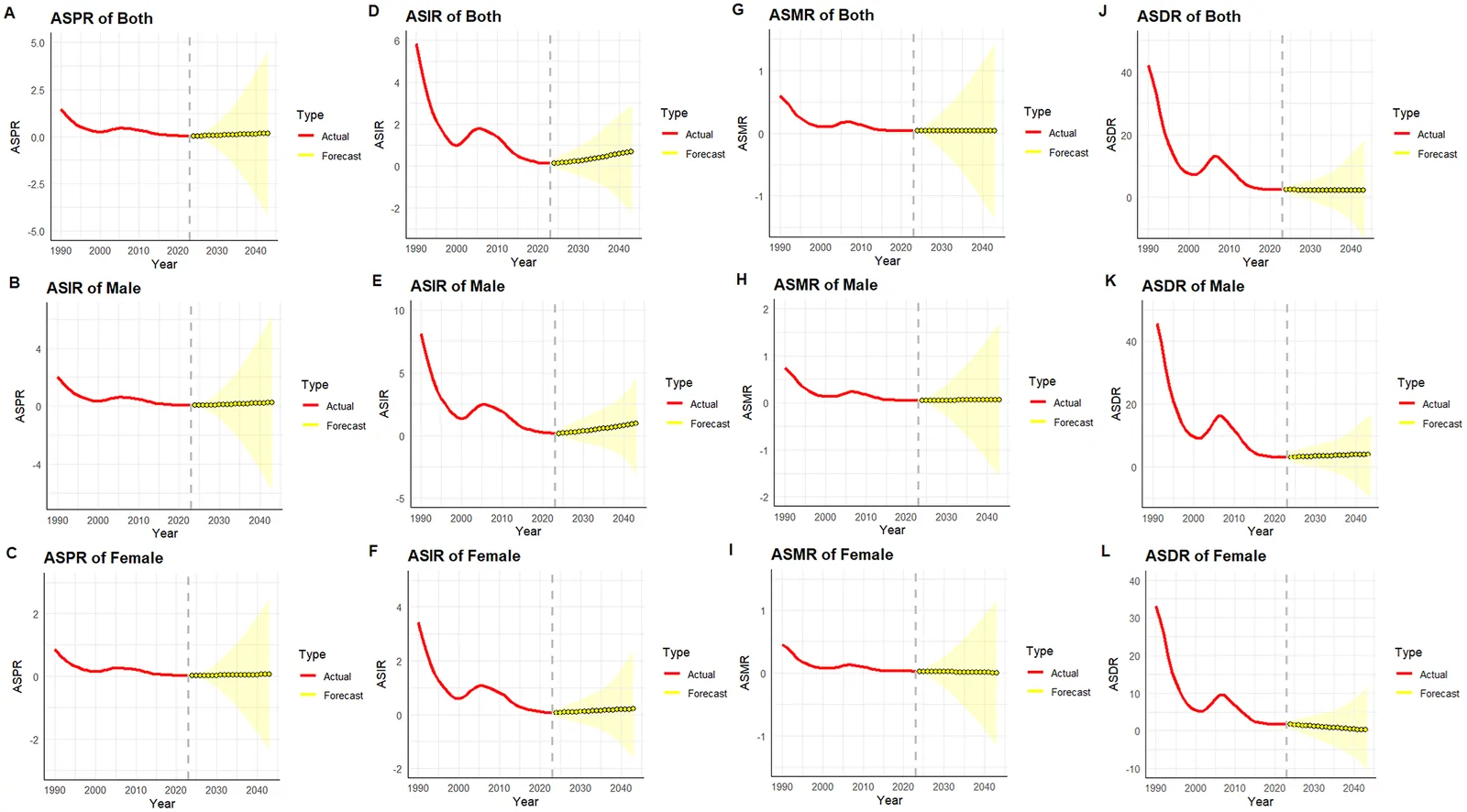
Projected trends of visceral leishmaniasis (VL) age-standardized rates in the G20 aggregate from 2024 to 2043 using the ARIMA model. (A–C) Prevalence; (D–F) Incidence; (G–I) Mortality; (J–L) DALYs. Panels A, D, G, and J show the overall trend for both sexes. Panels B, E, H, and K and C, F, I, and L stratify projections for males and females, respectively. Shaded regions represent 95% prediction intervals. The projections indicate a plateau or a mild increase in incidence and prevalence, with a persistent male-to-female disparity.

Projections suggested that ASPR and ASIR will remain relatively stable from 2024-2043, with a very subtle upward tick towards the end of the forecast period. The ASMR is projected to plateau, while the ASDR is expected to continue its gradual decline. Additionally, the sex disparity would persist, with all indicators consistently higher in males than in females (Fig 6A–6L and S8 Table). The MAPE of model backtesting was below 25%, this threshold confirms acceptable predictive performance for forecasting (S9 Table).

In addition, notable divergent trends in disease burden metrics were observed between the two extreme age subgroups: for children aged <5 years, prevalence and incidence rate will rise continuously throughout the forecast period, while both mortality and DALYs rate will steadily decline year by year; for the population aged ≥95 years, prevalence and incidence rate will follow a persistent downward trajectory and even turn negative in later years, mortality will remain flat without obvious fluctuations, and DALYs rate will experience a mild slow decline over the two decades. Additionally, sex disparity will persist across both age groups, with all burden indicators consistently higher in males than in females (S3 and S4 Figs and S10–S14 Tables).

## Discussion

This study provides the first comprehensive, multi-country assessment of VL burden across G20 countries from 1990 to 2023. The results demonstrated that while VL burden has declined substantially since 1990—with an overall reduction exceeding 90% for all age-standardized indicators—progress has been profoundly uneven, which is consistent with the overall progress of global VL prevention and control [3,18]. The core drivers of the overall reduction in VL burden across G20 countries are the implementation of vector control, early diagnosis, and standardized treatment strategies in key endemic regions under the tropical disease control programs led by the WHO [18,19]. At the national level, Saudi Arabia, Turkey, and India recorded the most substantial declines in all VL burden indicators, which is closely associated with the targeted control measures implemented in these three countries. As a core country in the high-prevalence VL belt of South Asia, India launched a national VL elimination program in 2000; this initiative has effectively curbed VL transmission by establishing a grassroots disease surveillance system, providing free first-line therapeutic drugs (e.g., miltefosine and amphotericin B), and conducting large-scale vector control campaigns against sandflies [20,21]. In contrast, Saudi Arabia and Turkey have drastically reduced disease burden caused by both local transmission and imported cases by improving entry quarantine systems, strengthening health management of mobile populations, and integrating targeted vector control with early case detection and treatment [22,23]. By contrast, Brazil now accounts for approximately 58% of all VL-related DALYs in the G20 bloc, a disproportionate share that warrants urgent international attention. VL transmission in Brazil is predominantly concentrated in impoverished northeastern regions characterized by insufficient health resources, frequent population mobility, and complex sandfly breeding habitats [24]. Furthermore, the coinfection of VL with human immunodeficiency virus (HIV) has further exacerbated the disease burden, which are key factors contributing to its relatively suboptimal control outcomes compared with other countries [25]. Additionally, although Argentina, China, the European Union, and several European countries have achieved significant reductions in VL burden, their control efforts have focused more on the surveillance and management of imported cases, which aligns with their intrinsic VL epidemiological profiles given the historically low endemicity of the disease in these regions [7,22].

Joinpoint regression analysis revealed the stage-specific characteristics of VL burden changes in G20 countries. Overall, the prevalence and incidence rates dropped sharply from 1990 to 2000, rebounded slightly between 2000 and 2004, stabilized from 2004 to 2007, and then continued to decrease. In contrast, the mortality and DALYs rates exhibited more fluctuating phases during the study period. This phased variation was closely linked to policy adjustments in global VL prevention and control as well as changes in external influencing factors. The marked decline from 1990 to 2000 was attributed to the WHO initial classification of VL as a priority NTD for elimination, which prompted increased investment in control efforts across countries [18,19]. The transient rebound between 2000–2004 may reflect a combination of factors, including potential waning of early control efforts, the emergence of drug resistance, or fluctuations in vector populations [23,26]. The renewed sharp decline after 2011 was driven by the development and application of novel therapeutic agents and the establishment of global partnerships for NTD elimination, which significantly enhanced the precision and implementation efficiency of control strategies [3,27].

Complementing pooled regression, this study also built country-specific segmented trend models for Brazil, India, Italy, and Turkey, linking disease burden changes to intervention timelines. Varied epidemiological cycles and strategies led to diverse outcomes: India achieved significant declines with comprehensive programs [21], while Turkey faced cross-border transmission offsetting vector control [23]. Italy’s canine interventions limited spread but not wildlife resurgences [28], and Brazil’s uneven surveillance hampered sustained reductions [24]. These findings underscore the inadequacy of single interventions, emphasizing the need for integrated, region-specific control for leishmaniasis.

Our finding of consistently higher burden in males and children under 5 years of age is consistent with established VL epidemiology [6,11]. Leishmania donovani exhibits greater invasiveness toward the immature immune systems of children, while males face a substantially higher risk of sandfly bites due to their more frequent outdoor activities [29,30]. As high-burden countries, Brazil and India reported far higher VL burden in children under 5 than in other demographic groups, which also suggests that these two nations should prioritize children in VL prevention and control efforts, strengthening health surveillance and preventive interventions for eligible pediatric populations. Furthermore, the observed upward tick in VL burden among those aged ≥95 years—albeit from a low baseline—is biologically and clinically intriguing. We hypothesize that this may reflect a combination of factors: (1) improved survival of older adults with chronic comorbidities, (2) age‑related immunosenescence increasing susceptibility to VL infection, and (3) late diagnosis due to atypical presentations in elderly populations, where nonspecific symptoms such as fever, weight loss, and fatigue are frequently misattributed to common geriatric syndromes (e.g., frailty, malnutrition, or malignancy) rather than VL—raising the possibility of diagnostic bias [31,32]. Critically, this finding also raises concerns about therapeutic vulnerability in this age group: age‑related pharmacokinetic changes, including reduced renal clearance of amphotericin B and altered drug distribution due to decreased lean body mass and serum albumin, may increase the risk of nephrotoxicity and treatment failure [33–35]. The clinical implication is that VL in the very elderly may be both under‑recognized and more difficult to treat safely. This is a testable hypothesis that warrants targeted surveillance and geriatric‑specific treatment protocols—recommendations not currently incorporated in any national VL control plan.

Decomposition analysis demonstrated that changes in VL burden across G20 countries from 1990 to 2023 resulted from the combined effects of population growth, population aging, and epidemiological changes, with the contribution of each factor varying substantially across different indicators and countries. For prevalence and incidence, population growth served as the primary positive contributor, whereas epidemiological changes exerted a negative effect. This finding indicates that although population expansion increased the potential baseline number of VL cases, the reduced transmission efficiency brought about by prevention and control measures was the core driver of the decline in case counts [12]. In contrast, shifts in mortality and DALYs were mainly positively driven by population aging and population growth, with epidemiological changes showing a pronounced negative contribution. This result reflects that demographic structural changes have elevated the potential risk of mortality and disability, while improvements in VL diagnosis and treatment have drastically reduced the disease’s fatality and disability rates [18,36]. Notably, variations in epidemiological rates in low-endemic regions such as Italy and the European Union exerted a positive impact on prevalence and incidence rates. This suggests that despite the absence of sustained local transmission in these areas, sporadic imported cases may still lead to a slight increase in regional disease burden [7,23]. Conversely, the strongly negative contribution of epidemiological rate changes to mortality in Saudi Arabia further confirms the marked effectiveness of its targeted prevention and control strategies in reducing disease fatality rates.

The projected stabilization and subtle increase in incidence and prevalence through 2043, coupled with persistent sex disparities, suggests that current control gains are fragile and that elimination targets remain contingent upon sustained investment. However, it is critical to emphasize that these ARIMA projections are purely data‑driven extrapolations of historical trends; they do not incorporate or test causal mechanisms. The subtle upward tick in the projections should be interpreted as a statistical signal warranting vigilance, not as a confirmed prediction of future increases. Several hypothetical drivers could, if they materialize, contribute to such a trend—including climate‑driven expansion of sandfly habitats [37], increased cross‑border population mobility [7], and changes in vector control coverage—but our data do not quantify their effects. Additionally, the stable mortality rate may be linked to the uneven allocation of diagnostic and treatment resources and insufficient accessibility to primary healthcare services in some high-burden countries, whereas the gradual decline in ASDR reflects that the long-term disability impacts caused by VL cannot be completely eliminated in the short term [4,30]. Furthermore, the persistent sex disparity also implies that future control strategies need to address gender-based disparities in healthcare accessibility and formulate targeted interventions [29,38]. Indeed, ARIMA models cannot distinguish whether the projected upward tick reflects underlying epidemiological forces, statistical noise, or the simple extrapolation of a decelerating decline. We therefore caution against over‑interpreting the projections as evidence that climate change or migration will necessarily reverse VL control gains. Rather, the projections serve as a warning that under a ‘business‑as‑usual’ scenario (i.e., continuation of current trends), the historical rate of decline may not be sustained, and that targeted surveillance and adaptive strategies are needed to detect and respond to any actual resurgence. Future research should integrate climate, land‑use, and migration data into multivariate forecasting models (e.g., ARIMAX or machine learning approaches) to test the contribution of these external drivers explicitly.

From a public health perspective, the key message of this study is not that VL has declined but that the decline has been inequitable and is at risk of reversal. Brazil’s disproportionate burden (ASDR: 30.54 vs. the next highest, India at 2.64) represents a failure of existing control strategies to achieve meaningful population impact. A reduction in Brazil’s ASDR by just 10%—from 30.54 to 27.49 per 100,000—would avert approximately 5,000 DALYs annually, equivalent to preventing 250 disability-adjusted life years per 100,000 population. This is a clinically and economically meaningful target. Our projections indicate that without intensified action, the G20 will not achieve the WHO 2030 elimination target of <1 per 100,000 persons in endemic countries. The subtle projected increase in incidence toward 2043 should not be dismissed as statistically trivial; even a 0.01 per 100,000 increase in Brazil translates to approximately 800 additional incident cases annually, each of which carries significant morbidity, mortality, and economic cost. We therefore recommend that the G20 health ministers prioritize a multilateral VL elimination fund, with Brazil as the first recipient, modeled on the successful ASEAN malaria elimination financing mechanism.

The primary strength of this study lies in its longitudinal, comparative design across a diverse group of countries with varying VL epidemiology. The joint application of joinpoint regression, decomposition analysis, and validated ARIMA models provides a comprehensive analytical framework that is novel in the VL literature. However, several limitations warrant critical reflection. First, the ecological design precludes causal inference; the associations we report (e.g., between Brazil’s poverty and VL burden) are suggestive but not definitive. Individual-level data on socioeconomic status, housing conditions, and access to healthcare would be necessary to establish causality. Second, the research data were derived from the GBD 2023 database, whose estimates are based on model integration and may thus deviate to some extent from the actual surveillance data of individual countries. Moreover, the GBD database only provides complete VL data for 10 out of the 20 G20 member states; the data gaps in the remaining countries may have exerted a certain impact on the overall analytical results. Third, when analyzing the driving factors, this study only considered population growth, population aging, and variations in epidemiological rates, while excluding more complex social and environmental factors such as climate change, socioeconomic development, health resource allocation vector control policy scaling, and cross-border mobility shifts. Therefore, the interpretation of the mechanisms underlying VL burden changes remains to be further refined. Fourth, the exclusion of nine G20 member states and Russia due to missing data introduces selection bias; our results are only generalizable to the 10 countries with complete estimates, not to the G20 as a whole. This is a non-trivial limitation that we explicitly acknowledge, and future work should prioritize completing VL burden estimates for all G20 nations through enhanced surveillance and modeling collaborations. Finally, the projections generated by the ARIMA model were solely based on historical time-series data, without accounting for sudden factors such as future adjustments to prevention and control strategies, the development of novel drugs, and public health emergencies. Thus, the predicted results need to be dynamically adjusted in light of actual circumstances.

## Conclusions

In summary, while VL burden has declined dramatically across G20 countries, this progress is fragile, inequitable, and threatened by demographic and environmental changes. Brazil represents a clear priority for intensified action. Our projections serve as a warning that without sustained political will and international cooperation, the gains of the past three decades may be partially undone. The G20’s leadership—both financial and political—is essential to achieving the 2030 elimination goals and preventing a potential resurgence. This study also raises several questions that future research should address: (1) How do subnational variations within Brazil, India, and other G20 countries affect national burden estimates? Subnational analyses using the GBD subnational data are urgently needed. (2) What is the contribution of climate change to VL expansion in temperate G20 countries? Predictive models incorporating climate, land use, and vector distributions would improve projection accuracy. (3) Can machine learning approaches outperform ARIMA in predicting VL trends given the non-linearities introduced by policy shifts and climate variability? We are currently exploring these alternatives. (4) What are the economic costs of VL in G20 countries, and how do they compare to the costs of elimination? Cost-effectiveness analyses are needed to justify investment.

## Supporting information

S1 Table Joinpoint regression analysis of age-standardized prevalence rate, age-standardized incidence rate, age-standardized mortality rate, and age-standardized DALYs rate of visceral leishmaniasis in G20 from 1990 to 2023. (DOCX)

S2 Table Joinpoint regression analysis of age-standardized prevalence rate, age-standardized incidence rate, age-standardized mortality rate, and age-standardized DALYs rate of visceral leismaniasis in Brazil, India, Italy and Turkey from 1990 to 2023. (DOCX)

S3 Table Trends in sex- and age-specific prevalence, incidence, mortality, and disability-adjusted life years of visceral leishmaniasis in G20 countries, Brazil, India, Italy, and Turkey (1990 and 2023). (DOCX)

S4 Table Trends in visceral leishmaniasis prevalence in G20 countries, decomposed by population aging, population growth and epidemiological changes, 1990–2023. (DOCX)

S5 Table Trends in visceral leishmaniasis incidence in G20 countries, decomposed by population aging, population growth and epidemiological changes, 1990–2023. (DOCX)

S6 Table Trends in visceral leishmaniasis mortality in G20 countries, decomposed by population aging, population growth and epidemiological changes, 1990–2023. (DOCX)

S7 Table Trends in visceral leishmaniasis DALYs in G20 countries, decomposed by population aging, population growth and epidemiological changes, 1990–2023. (DOCX)

S8 Table Predicted all-age age-standardized prevalence rate, age-standardized incidence rate, age-standardized mortality rate, and age-standardized DALYs rate of visceral leishmaniasis in G20 from 2024 to 2043. (DOCX)

S9 Table Back-testing analysis of all-age age-standardized prevalence rate, age-standardized incidence rate, age-standardized mortality rate, and age-standardized DALYs rate burden prediction for visceral leishmaniasis in G20, 2014–2023. (DOCX)

S10 Table Predicted prevalence rate, incidence rate, mortality rate, and DALYs rate of visceral leishmaniasis aged <5 years in G20 from 2024 to 2043. (DOCX)

S11 Table Predicted prevalence rate, incidence rate, mortality rate, and DALYs rate of visceral leishmaniasis aged ≥95 years in G20 from 2024 to 2043. (DOCX)

S12 Table Back-testing analysis of prevalence rate, incidence rate, mortality rate, and DALYs rate burden prediction for visceral leishmaniasis aged <5 years in G20, 2014–2023. (DOCX)

S13 Table Back-testing analysis of age-standardized prevalence rate, age-standardized incidence rate, age-standardized mortality rate, and age-standardized DALYs rate burden prediction for visceral leishmaniasis aged ≥95 years in G20, 2014–2023. (DOCX)

S14 Table Optimal Parameters of Gender-Stratified ARIMA Models Selected by the auto.arima() Function. (DOCX)

S1 Fig Heatmap of age-standardized rates for visceral leishmaniasis (VL) in G20 countries, 2023. (A) Prevalence; (B) Incidence; (C) Mortality; (D) Disability-adjusted life years (DALYs). Color intensity reflects the magnitude of the rate, with darker shades indicating higher burden. This visualization facilitates direct comparison across countries and indicators.
(DOCX)

S2 Fig Joinpoint regression analysis of visceral leishmaniasis (VL) age-standardized rates by both sex in Brazil, India, Italy and Turkey aggregate, 1990–2023. (A) Prevalence; (B) Incidence; (C) Mortality; (D) Disability-adjusted life years (DALYs). Solid lines represent fitted trend segments identified by the joinpoint model. The annual percentage change (APC) and its significance (*P < 0.05) are displayed for each segment. The distinct trend phases, including a notable decline post-2011, are clearly delineated.
(DOCX)

S3 Fig Projected age-standardized rate trends of visceral leishmaniasis (VL) in the < 5 years age group across the aggregated G20 economies, 2024–2043, based on the ARIMA model. (A–C) Prevalence; (D–F) Incidence; (G–I) Mortality; (J–L) DALYs. Panels A, D, G, and J show the overall trend for both sexes. Panels B, E, H, and K and C, F, I, and L stratify projections for males and females, respectively. Shaded regions represent 95% prediction intervals.
(DOCX)

S4 Fig Projected age-standardized rate trends of visceral leishmaniasis (VL) in the ≥ 95 years age group across the aggregated G20 economies, 2024–2043, based on the ARIMA model. (A–C) Prevalence; (D–F) Incidence; (G–I) Mortality; (J–L) DALYs. Panels A, D, G, and J show the overall trend for both sexes. Panels B, E, H, and K and C, F, I, and L stratify projections for males and females, respectively. Shaded regions represent 95% prediction intervals.
(DOCX)

S1 File The complete R code for all analyses and figures in our study. (ZIP)
