# Supplementary material for: Epidemiological transitions of Visceral Leishmaniasis in G20 countries: A 33-year retrospective joinpoint and demographic decomposition analysis with 20-year ARIMA projections
Source: PLoS Negl Trop Dis. 2026 Sep 16;20(9):e0014714. doi: 10.1371/journal.pntd.0014714 (PMC13580953; doi:10.1371/journal.pntd.0014714)
Supplement: Supplementary file 1 [file pntd.0014714.s001.docx]

**S1 Table:** Joinpoint regression analysis of age-standardized prevalence rate, age-standardized incidence rate, age-standardized mortality rate, and age-standardized DALYs rate of visceral leishmaniasis in G20 from 1990 to 2023.

| **Trends** | **ASPR** | | | | | | | | | | |
| --- | --- | --- | --- | --- | --- | --- | --- | --- | --- | --- | --- |
|  | **Both** | | |  | **Male** | | |  | **Female** | | |
|  | **Year** | **APC, % (95%CI)** | ***P*** |  | **Year** | **APC, % (95%CI)** | ***P*** |  | **Year** | **APC, % (95%CI)** | ***P*** |
| Trend 1 | 1990-2000 | -16.18(-17.73,-14.60) | <0.001 |  | 1990-2000 | -16.27(-17.80,-14.72) | <0.001 |  | 1990-2000 | -16.05(-16.77,-15.32) | <0.001 |
| Trend 2 | 2000-2004 | 17.65(13.11,22.37) | <0.001 |  | 2000-2004 | 17.68(13.30,22.23) | <0.001 |  | 2000-2004 | 17.64(15.27,20.05) | <0.001 |
| Trend 3 | 2004-2007 | 0.83(-5.48,7.57) | 0.792 |  | 2004-2007 | 0.80(-5.46,7.48) | 0.798 |  | 2004-2007 | 0.70(-2.41,3.90) | 0.646 |
| Trend 4 | 2007-2011 | -8.64(-11.58,-5.60) | <0.001 |  | 2007-2011 | -8.64(-11.51,-5.68) | <0.001 |  | 2007-2011 | -8.00(-9.40,-6.56) | <0.001 |
| Trend 5 | 2011-2023 | -19.69(-20.33,-19.03) | <0.001 |  | 2011-2023 | -19.74(-20.38,-19.09) | <0.001 |  | 2011-2016 | -21.15(-22.26,-20.02) | <0.001 |
| Trend 6 | --- | --- | --- |  | --- | --- | --- |  | 2016-2023 | -17.06(-18.01,-16.10) | <0.001 |
| AAPC (95%CI) | 1990-2023 | -11.63(-12.51,-10.74) | <0.001 |  | 1990-2023 | -11.69(-12.55,-10.81) | <0.001 |  | 1990-2023 | -11.17(-11.66,-10.68) | <0.001 |
| **Trends** | **ASIR** | | | | | | | | | | |
|  | **Both** | | |  | **Male** | | |  | **Female** | | |
|  | **Year** | **APC, % (95%CI)** | ***P*** |  | **Year** | **APC, % (95%CI)** | ***P*** |  | **Year** | **APC, % (95%CI)** | ***P*** |
| Trend 1 | 1990-2000 | -16.18(-17.73,-14.59) | <0.001 |  | 1990-2000 | -16.27(-17.80,-14.72) | <0.001 |  | 1990-2000 | -16.05(-16.77,-15.32) | <0.001 |
| Trend 2 | 2000-2004 | 17.65(13.10,22.38) | <0.001 |  | 2000-2004 | 17.68(13.30,22.23) | <0.001 |  | 2000-2004 | 17.64(15.26,20.06) | <0.001 |
| Trend 3 | 2004-2007 | 0.83 (-5.49,7.57) | 0.792 |  | 2004-2007 | 0.80(-5.46,7.48) | 0.798 |  | 2004-2007 | 0.70(-2.42,3.92) | 0.647 |
| Trend 4 | 2007-2011 | -8.64(-11.58,-5.60) | <0.001 |  | 2007-2011 | -8.64(-11.51,-5.68) | <0.001 |  | 2007-2011 | -7.99(-9.41,-6.55) | <0.001 |
| Trend 5 | 2011-2023 | -19.68(-20.33,-19.03) | <0.001 |  | 2011-2023 | -19.74(-20.38,-19.09) | <0.001 |  | 2011-2016 | -21.15(-22.26,-20.02) | <0.001 |
| Trend 6 | --- | --- | --- |  | --- | --- | --- |  | 2016-2023 | -17.05(-18.00,-16.09) | <0.001 |
| AAPC (95%CI) | 1990-2023 | -11.63(-12.52,-10.74) | <0.001 |  | 1990-2023 | -11.69(-12.55,-10.81) | <0.001 |  | 1990-2023 | -11.17(-11.66,-10.68) | <0.001 |
| **Trends** | **ASMR** | | | | | | | | | | |
|  | **Both** | | |  | **Male** | | |  | **Female** | | |
|  | **Year** | **APC, % (95%CI)** | ***P*** |  | **Year** | **APC, % (95%CI)** | ***P*** |  | **Year** | **APC, % (95%CI)** | ***P*** |
| Trend 1 | 1990-1999 | -17.66(-18.39,-16.92) | <0.001 |  | 1990-1999 | -17.32(-18.06,-16.57) | <0.001 |  | 1990-1999 | -18.29(-19.04,-17.53) | <0.001 |
| Trend 2 | 1999-2002 | -3.16(-12.10,6.70) | 0.494 |  | 1999-2002 | -2.93(-11.99,7.05) | 0.530 |  | 1999-2002 | -3.40(-12.46,6.60) | 0.469 |
| Trend 3 | 2002-2006 | 17.40(11.83,23.25) | <0.001 |  | 2002-2006 | 17.12(11.59,22.92) | <0.001 |  | 2002-2006 | 17.25(11.52,23.28) | <0.001 |
| Trend 4 | 2006-2010 | -8.05(-12.39,-3.51) | 0.002 |  | 2006-2010 | -7.86(-12.22,-3.29) | 0.002 |  | 2006-2009 | -5.86(-14.78,4.00) | 0.218 |
| Trend 5 | 2010-2016 | -17.65(-19.39,-15.87) | <0.001 |  | 2010-2016 | -17.51(-19.28,-15.71) | <0.001 |  | 2009-2016 | -17.27(-18.61,-15.91) | <0.001 |
| Trend 6 | 2016-2023 | -3.13(-4.32,-1.93) | <0.001 |  | 2016-2023 | -3.33(-4.53,-2.12) | <0.001 |  | 2016-2023 | -3.01(-4.21,-1.80) | <0.001 |
| AAPC (95%CI) | 1990-2023 | -8.49(-9.60,-7.36) | <0.001 |  | 1990-2023 | -8.38(-9.51,-7.24) | <0.001 |  | 1990-2023 | -8.71(-9.97,-7.43) | <0.001 |
| **Trends** | **ASDR** | | | | | | | | | | |
|  | **Both** | | |  | **Male** | | |  | **Female** | | |
|  | **Year** | **APC, % (95%CI)** | ***P*** |  | **Year** | **APC, % (95%CI)** | ***P*** |  | **Year** | **APC, % (95%CI)** | ***P*** |
| Trend 1 | 1990-1999 | -17.55(-18.29,-16.80) | <0.001 |  | 1990-1999 | -17.14(-17.89,-16.38) | <0.001 |  | 1990-1999 | -18.30(-19.05,-17.54) | <0.001 |
| Trend 2 | 1999-2002 | -3.56(-12.65,6.47) | 0.450 |  | 1999-2002 | -3.30(-12.44,6.80) | 0.486 |  | 1999-2002 | -4.05(-13.13,5.97) | 0.392 |
| Trend 3 | 2002-2006 | 17.46(11.80,23.41) | <0.001 |  | 2002-2006 | 16.99(11.38,22.88) | <0.001 |  | 2002-2006 | 18.37(12.51,24.55) | <0.001 |
| Trend 4 | 2006-2010 | -8.11(-12.53,-3.48) | 0.002 |  | 2006-2010 | -7.90(-12.32,-3.25) | 0.003 |  | 2006-2010 | -8.49(-12.99,-3.76) | 0.002 |
| Trend 5 | 2010-2016 | -18.16(-19.92,-16.36) | <0.001 |  | 2010-2016 | -17.89(-19.66,-16.08) | <0.001 |  | 2010-2016 | -18.68(-20.46,-16.87) | <0.001 |
| Trend 6 | 2016-2023 | -3.28(-4.49,-2.06) | <0.001 |  | 2016-2023 | -3.48(-4.70,.25) | <0.001 |  | 2016-2023 | -2.93(-4.15,-1.70) | <0.001 |
| AAPC (95%CI) | 1990-2023 | -8.62(-9.76,-7.47) | <0.001 |  | 1990-2023 | -8.48(-9.62,-7.33) | <0.001 |  | 1990-2023 | -8.89(-10.04,-7.73) | <0.001 |
