# Supplementary material for: Epidemiological transitions of Visceral Leishmaniasis in G20 countries: A 33-year retrospective joinpoint and demographic decomposition analysis with 20-year ARIMA projections
Source: PLoS Negl Trop Dis. 2026 Sep 16;20(9):e0014714. doi: 10.1371/journal.pntd.0014714 (PMC13580953; doi:10.1371/journal.pntd.0014714)
Supplement: Supplementary file 2 [file pntd.0014714.s002.docx]

**S2 Table:** Joinpoint regression analysis of age-standardized prevalence rate, age-standardized incidence rate, age-standardized mortality rate, and age-standardized DALYs rate of visceral leismaniasis in Brazil, India, Italy and Turkey from 1990 to 2023.

| **Trends** | **ASPR** | | | | | | | | | | | | | | |  |
| --- | --- | --- | --- | --- | --- | --- | --- | --- | --- | --- | --- | --- | --- | --- | --- | --- |
|  | **Brazil** | | |  | **India** | | |  | **Italy** | | |  | **Turkey** | | |  |
|  | **Year** | **APC, % (95%CI)** | ***P*** |  | **Year** | **APC, % (95%CI)** | ***P*** |  | **Year** | **APC, % (95%CI)** | ***P*** |  | **Year** | **APC, % (95%CI)** | ***P*** |  |
| Trend 1 | 1990-1993 | 8.90  (6.46,11.39) | <0.001 |  | 1990-2000 | -20.13  (-21.19,-19.05) | <0.001 |  | 1990-1994 | 10.89  (9.27,12.54) | <0.001 |  | 1990-1997 | -19.16  (-21.51,-16.74) | <0.001 |  |
| Trend 2 | 1993-1996 | 1.16  (-2.15,4.58) | 0.476 |  | 2000-2004 | 21.95  (18.33,25.67) | <0.001 |  | 1994-2000 | 2.08  (1.55,2.60) | <0.001 |  | 1997-2000 | -59.45  (-62.06,-56.66) | <0.001 |  |
| Trend 3 | 1996-2000 | -4.13  (-4.99,-3.26) | <0.001 |  | 2004-2007 | 0.21  (-4.38,5.03) | 0.925 |  | 2000-2005 | -5.20  (-5.90,-4.50) | <0.001 |  | 2000-2023 | -3.27  (-3.76,-2.77) | <0.001 |  |
| Trend 4 | 2000-2014 | -1.44  (-1.51,-1.37) | <0.001 |  | 2007-2011 | -8.94  (-10.99,-6.83) | <0.001 |  | 2005-2010 | -14.41  (-15.24,-13.57) | <0.001 |  | --- | --- | --- |  |
| Trend 5 | 2014-2017 | -5.74  (-8.02,-3.41) | <0.001 |  | 2011-2023 | -24.99  (-25.64,-24.34) | <0.001 |  | 2010-2019 | -5.61  (-6.06,-5.15) | <0.001 |  | --- | --- | --- |  |
| Trend 6 | 2017-2023 | -12.07  (-12.62,-11.51) | <0.001 |  | --- | --- | --- |  | 2019-2023 | 0.80  (-2.02,3.70) | 0.562 |  | --- | --- | --- |  |
| AAPC (95%CI) | 1990-2023 | -3.07  (-3.48,-2.66) | <0.001 |  | 1990-2023 | -14.77  (-15.41,-14.12) | <0.001 |  | 1990-2023 | -2.97  (-3.39,-2.56) | <0.001 |  | 1990-2023 | -13.96  (-14.73,-13.18) | <0.001 |  |
| **Trends** | **ASIR** | | | | | | | | | | | | | | |  |
|  | **Brazil** | | |  | **India** | | |  | **Italy** | | |  | **Turkey** | | |  |
|  | **Year** |  | ***P*** |  | **Year** | **APC, % (95%CI)** | ***P*** |  | **Year** | **APC, % (95%CI)** | ***P*** |  | **Year** | **APC, % (95%CI)** | ***P*** |  |
| Trend 1 | 1990-1993 | 8.90  (6.46,11.40) | <0.001 |  | 1990-2000 | -20.13  (-21.19,-19.05) | <0.001 |  | 1990-1994 | 10.89  (9.27,12.54) | <0.001 |  | 1990-1997 | -19.16  (-21.51,-16.74) | <0.001 |  |
| Trend 2 | 1993-1996 | 1.16  (-2.16,4.59) | 0.476 |  | 2000-2004 | 21.95  (18.32,25.68) | <0.001 |  | 1994-2000 | 2.08  (1.55,2.60) | <0.001 |  | 1997-2000 | -59.45  (-62.06,-56.66) | <0.001 |  |
| Trend 3 | 1996-2000 | -4.13  (-4.99,-3.26) | <0.001 |  | 2004-2007 | 0.21  (-4.39,5.04) | 0.925 |  | 2000-2005 | -5.20  (-5.90,-4.50) | <0.001 |  | 2000-2023 | -3.27  (-3.76,-2.77) | <0.001 |  |
| Trend 4 | 2000-2014 | -1.44  (-1.51,-1.37) | <0.001 |  | 2007-2011 | -8.94  (-11.00,-6.83) | <0.001 |  | 2005-2010 | -14.41  (-15.24,-13.57) | <0.001 |  | --- | --- | --- |  |
| Trend 5 | 2014-2017 | -5.74  (-8.02,-3.40) | <0.001 |  | 2011-2023 | -24.99  (-25.64,-24.34) | <0.001 |  | 2010-2019 | -5.61  (-6.06,-5.15) | <0.001 |  | --- | --- | --- |  |
| Trend 6 | 2017-2023 | -12.06  (-12.62,-11.51) | <0.001 |  | --- | --- | --- |  | 2019-2023 | 0.80  (-2.02,3.70) | 0.562 |  | --- | --- | --- |  |
| AAPC (95%CI) | 1990-2023 | -3.07  (-3.49,-2.66) | <0.001 |  | 1990-2023 | -14.77  (-15.41,-14.11) | <0.001 |  | 1990-2023 | -2.97  (-3.39,-2.56) | <0.001 |  | 1990-2023 | -13.96  (-14.73,-13.18) | <0.001 |  |
| **Trends** | **ASMR** | | | | | | | | | | | | | | |  |
|  | **Brazil** | | |  | **India** | | |  | **Italy** | | |  | **Turkey** | | |  |
|  | **Year** | **APC, % (95%CI)** | ***P*** |  | **Year** | **APC, % (95%CI)** | ***P*** |  | **Year** | **APC, % (95%CI)** | ***P*** |  | **Year** | **APC, % (95%CI)** | ***P*** |  |
| Trend 1 | 1990-1995 | 6.61  (4.88,8.36) | <0.001 |  | 1990-1999 | -22.05  (-23.01,-21.08) | <0.001 |  | 1990-1992 | 21.27  (12.09,31.21) | <0.001 |  | 1990-1995 | -13.16  (-16.32,-9.88) | <0.001 |  |
| Trend 2 | 1995-2002 | -3.69  (-4.86,-2.51) | <0.001 |  | 1999-2002 | -3.89  (-16.27,10.31) | 0.551 |  | 1992-2002 | 1.29  (0.56,2.03) | 0.001 |  | 1995-2000 | -51.54  (-54.00,-48.95) | <0.001 |  |
| Trend 3 | 1902-2008 | 1.13  (-0.48,2.76) | 0.157 |  | 2002-2006 | 22.11  (14.05,30.74) | <0.001 |  | 2002-2011 | -12.37  (-13.11,-11.63) | <0.001 |  | 2000-2013 | -1.79  (-2.73,-0.85) | <0.001 |  |
| Trend 4 | 2008-2013 | -6.28  (-8.39,-4.12) | <0.001 |  | 2006-2010 | -8.73  (-14.69,-2.35) | 0.011 |  | 2011-2023 | 0.47  (0.00,0.95) | 0.051 |  | 2013-2023 | -6.36  (-7.52,-5.19) | <0.001 |  |
| Trend 5 | 2013-2017 | 2.18  (-1.41,5.91) | 0.221 |  | 2010-2017 | -24.36  (-26.06,-22.62) | <0.001 |  | --- | --- | --- |  | --- | --- | --- |  |
| Trend 6 | 2017-2023 | -0.96  (-2.14,0.25) | 0.112 |  | 2017-2023 | -5.99  (-8.10,-3.83) | --- |  | --- | --- | --- |  | --- | --- | --- |  |
| AAPC (95%CI) | 1990-2023 | -0.52  (-1.21,0.18) | 0.146 |  | 1990-2023 | -12.09  (-13.59,-10.56) | <0.001 |  | 1990-2023 | -1.85  (-2.41,-1.30) | <0.001 |  | 1990-2023 | -14.63  (-15.52,-13.73) | <0.001 |  |
| **Trends** | **ASDR** | | | | | | | | | | | | | | |  |
|  | **Brazil** | | |  | **India** | | |  | **Italy** | | |  | **Turkey** | | |  |
|  | **Year** | **APC, % (95%CI)** | ***P*** |  | **Year** | **APC, % (95%CI)** | ***P*** |  | **Year** | **APC, % (95%CI)** | ***P*** |  | **Year** | **APC, % (95%CI)** | ***P*** |  |
| Trend 1 | 1990-1995 | 6.60  (4.84,8.38) | <0.001 |  | 1990-2000 | -21.84  (-22.71,-20.95) | <0.001 |  | 1990-1992 | 21.41  (12.41,31.14) | <0.001 |  | 1990-1994 | -4.79  (-13.01,4.21) | <0.001 |  |
| Trend 2 | 1995-2002 | -3.54  (-4.73,-2.34) | <0.001 |  | 2000-2007 | 13.51  (10.75,16.33) | <0.001 |  | 1992-2002 | 1.09  (0.37,1.82) | <0.001 |  | 1994-2000 | -47.32  (-50.56,-43.87) | <0.001 |  |
| Trend 3 | 2002-2008 | 0.95  (-0.67,2.61) | 0.235 |  | 2007-2011 | -13.62  (-19.60,-7.20) | 0.925 |  | 2002-2011 | -12.48  (-13.21,-11.74) | <0.001 |  | 2000-2023 | -3.97  (-4.56,-3.37) | <0.001 |  |
| Trend 4 | 2008-2013 | -6.39  (-8.53,-4.20) | <0.001 |  | 2011-2017 | -25.46  (-27.80,-23.04) | <0.001 |  | 2011-2023 | 0.33  (-0.14,0.80) | <0.001 |  | --- | --- | --- |  |
| Trend 5 | 2013-2017 | 1.90  (-1.74,5.66) | 0.290 |  | 2017-2023 | -5.41  (-7.68,-3.08) | <0.001 |  | --- | --- | --- |  | --- | --- | --- |  |
| Trend 6 | 2017-2023 | -1.09  (-2.30,0.12) | 0.075 |  | --- | --- | --- |  | --- | --- | --- |  | --- | --- | --- |  |
| AAPC (95%CI) | 1990-2023 | -0.59  (-1.30,0.11) | 0.100 |  | 1990-2023 | -12.11  (-13.17,-11.03) | <0.001 |  | 1990-2023 | -1.99  (-2.53,-1.44) | <0.001 |  | 1990-2023 | -13.99  (-15.33,-12.63) | <0.001 |  |
