# Supplementary material for: Epidemiological transitions of Visceral Leishmaniasis in G20 countries: A 33-year retrospective joinpoint and demographic decomposition analysis with 20-year ARIMA projections
Source: PLoS Negl Trop Dis. 2026 Sep 16;20(9):e0014714. doi: 10.1371/journal.pntd.0014714 (PMC13580953; doi:10.1371/journal.pntd.0014714)
Supplement: Supplementary file 3 [file pntd.0014714.s003.docx]

**S3 Table** Trends in sex- and age-specific prevalence, incidence, mortality, and disability-adjusted life years of visceral leishmaniasis in G20 countries, Brazil, India, Italy, and Turkey (1990 and 2023).

| **Index** | **Prevalence Rate** | | | |
| --- | --- | --- | --- | --- |
|  | **1990** | | **2023** | |
|  | **Male** | **Female** | **Male** | **Female** |
| **G20** | | | | |
| <5 years | 6.016419781 | 3.198551962 | 0.118618944 | 0.065185931 |
| 5-9 years | 6.027898264 | 2.366403196 | 0.095073495 | 0.03900347 |
| 10-14 years | 2.890487574 | 0.960928719 | 0.053730885 | 0.019276818 |
| 15-19 years | 1.474068903 | 0.464303931 | 0.034682607 | 0.012014594 |
| 20-24 years | 1.039858943 | 0.340874416 | 0.025927336 | 0.009247837 |
| 25-29 years | 1.079037066 | 0.390239139 | 0.026199819 | 0.009988508 |
| 30-34 years | 1.028241876 | 0.408210349 | 0.02268473 | 0.009367394 |
| 35-39 years | 0.864266651 | 0.376124484 | 0.018879748 | 0.008755864 |
| 40-44 years | 0.696346172 | 0.324209664 | 0.015973421 | 0.00798298 |
| 45-49 years | 0.556923448 | 0.260067234 | 0.011287901 | 0.005919612 |
| 50-54 years | 0.445826352 | 0.212417706 | 0.008979835 | 0.004959907 |
| 55-59 years | 0.43245957 | 0.207992201 | 0.008667958 | 0.004845088 |
| 60-64 years | 0.442594118 | 0.192991649 | 0.009352384 | 0.005074805 |
| 65-69 years | 0.446509746 | 0.179328183 | 0.009476228 | 0.004961712 |
| 70-74 years | 0.389891754 | 0.154039302 | 0.009357464 | 0.004797853 |
| 75-79 years | 0.366563471 | 0.120096043 | 0.009664654 | 0.004835886 |
| 80-84 years | 0.444446868 | 0.112051453 | 0.009818257 | 0.004618015 |
| 85-89 years | 0.413817225 | 0.091700156 | 0.010055036 | 0.00444745 |
| 90-94 years | 0.470734912 | 0.086875914 | 0.012744206 | 0.004859934 |
| 95+ years | 0.572890403 | 0.091711009 | 0.023420174 | 0.006161925 |
| **Brazil** | | | | |
| <5 years | 2.826264671 | 1.529261309 | 1.082596246 | 0.587344649 |
| 5-9 years | 2.668684881 | 1.073707643 | 0.93351606 | 0.37425328 |
| 10-14 years | 1.468200587 | 0.517205672 | 0.527021823 | 0.184882806 |
| 15-19 years | 0.906575675 | 0.308500006 | 0.3386895 | 0.115385948 |
| 20-24 years | 0.646787827 | 0.227316903 | 0.249190554 | 0.087308554 |
| 25-29 years | 0.665563119 | 0.246117908 | 0.266381558 | 0.09851567 |
| 30-34 years | 0.626078945 | 0.250476363 | 0.25699551 | 0.102580018 |
| 35-39 years | 0.521434217 | 0.233552799 | 0.215874733 | 0.096569401 |
| 40-44 years | 0.422515206 | 0.203736331 | 0.169026218 | 0.081602831 |
| 45-49 years | 0.329561921 | 0.167907135 | 0.127617918 | 0.065284028 |
| 50-54 years | 0.287381397 | 0.154219557 | 0.110098658 | 0.059219172 |
| 55-59 years | 0.291280126 | 0.15540243 | 0.111451735 | 0.060556903 |
| 60-64 years | 0.315154052 | 0.162183136 | 0.117896442 | 0.061856761 |
| 65-69 years | 0.355337443 | 0.174295291 | 0.126863355 | 0.064367885 |
| 70-74 years | 0.369910116 | 0.181236568 | 0.130385983 | 0.065842548 |
| 75-79 years | 0.388075491 | 0.186146882 | 0.138251484 | 0.067106927 |
| 80-84 years | 0.413449141 | 0.189770032 | 0.150023716 | 0.069636421 |
| 85-89 years | 0.461305833 | 0.196415116 | 0.168543441 | 0.074158861 |
| 90-94 years | 0.504264169 | 0.215986532 | 0.222931474 | 0.085324465 |
| 95+ years | 0.684508166 | 0.280972065 | 0.281309669 | 0.097403661 |
| **India** | | | | |
| <5 years | 17.78487227 | 9.523265328 | 0.131183341 | 0.070883233 |
| 5-9 years | 17.31515249 | 6.894879216 | 0.12073472 | 0.048904749 |
| 10-14 years | 8.918293935 | 3.020027902 | 0.067260481 | 0.023873234 |
| 15-19 years | 5.259981982 | 1.70145568 | 0.042025833 | 0.014199225 |
| 20-24 years | 3.958104174 | 1.331386119 | 0.029604687 | 0.010108691 |
| 25-29 years | 4.223594276 | 1.558930799 | 0.028710881 | 0.010257295 |
| 30-34 years | 4.11760061 | 1.660220615 | 0.025736575 | 0.009907647 |
| 35-39 years | 3.639116345 | 1.639721978 | 0.021668651 | 0.009488459 |
| 40-44 years | 2.900412091 | 1.400714238 | 0.017462582 | 0.008241995 |
| 45-49 years | 2.201514479 | 1.096013734 | 0.01286806 | 0.006226579 |
| 50-54 years | 1.953130481 | 1.003055574 | 0.010695626 | 0.005395614 |
| 55-59 years | 1.940444191 | 1.020163985 | 0.010652053 | 0.005430709 |
| 60-64 years | 2.096157426 | 1.05063073 | 0.011771292 | 0.005837892 |
| 65-69 years | 2.311992445 | 1.138228717 | 0.013442711 | 0.006344157 |
| 70-74 years | 2.145103662 | 1.046060925 | 0.014162379 | 0.006393309 |
| 75-79 years | 2.306157278 | 1.016036663 | 0.013514746 | 0.005575073 |
| 80-84 years | 2.729216926 | 1.028663042 | 0.011207548 | 0.004275829 |
| 85-89 years | 2.91549785 | 1.103588324 | 0.009603216 | 0.003239959 |
| 90-94 years | 3.530045878 | 1.27992401 | 0.010593915 | 0.00285711 |
| 95+ years | 3.94995708 | 1.433702517 | 0.012995975 | 0.002960864 |
| **Italy** | | | | |
| <5 years | 0.380487806 | 0.205286832 | 0.139855101 | 0.075165804 |
| 5-9 years | 0.368317504 | 0.147082683 | 0.133214312 | 0.053299489 |
| 10-14 years | 0.202976924 | 0.071138 | 0.074320087 | 0.026116603 |
| 15-19 years | 0.123488241 | 0.042195127 | 0.048110767 | 0.016364058 |
| 20-24 years | 0.089849193 | 0.031775737 | 0.037231254 | 0.01300399 |
| 25-29 years | 0.095272542 | 0.035829002 | 0.040723856 | 0.015013215 |
| 30-34 years | 0.092502719 | 0.03749304 | 0.039807552 | 0.015820567 |
| 35-39 years | 0.076766767 | 0.034767742 | 0.033382448 | 0.014896273 |
| 40-44 years | 0.059955626 | 0.029358613 | 0.026418919 | 0.012913496 |
| 45-49 years | 0.043502376 | 0.022850896 | 0.019716636 | 0.010279983 |
| 50-54 years | 0.03739508 | 0.020870704 | 0.017010567 | 0.009463139 |
| 55-59 years | 0.039152331 | 0.021853323 | 0.01753397 | 0.009826663 |
| 60-64 years | 0.04241212 | 0.022814292 | 0.019331801 | 0.010588339 |
| 65-69 years | 0.045988343 | 0.023622857 | 0.021367316 | 0.011291909 |
| 70-74 years | 0.0466262 | 0.023936294 | 0.021047834 | 0.011181577 |
| 75-79 years | 0.04684448 | 0.022826257 | 0.021088025 | 0.011066331 |
| 80-84 years | 0.049054744 | 0.022333092 | 0.021032415 | 0.01076348 |
| 85-89 years | 0.055305459 | 0.023494515 | 0.023072423 | 0.011315877 |
| 90-94 years | 0.069982603 | 0.026204458 | 0.029165647 | 0.012516024 |
| 95+ years | 0.094033012 | 0.031122574 | 0.036107447 | 0.013567733 |
| **Turkey** | | | | |
| <5 years | 4.818464377 | 2.608898565 | 0.041224586 | 0.022167625 |
| 5-9 years | 4.631414658 | 1.855691083 | 0.039023818 | 0.015618194 |
| 10-14 years | 2.58202109 | 0.906938849 | 0.021751069 | 0.007632422 |
| 15-19 years | 1.63826476 | 0.557660916 | 0.013804267 | 0.004693643 |
| 20-24 years | 1.255590349 | 0.437895628 | 0.010574091 | 0.003687145 |
| 25-29 years | 1.380528702 | 0.507332564 | 0.011621345 | 0.00427343 |
| 30-34 years | 1.342733843 | 0.532443204 | 0.011309041 | 0.004485892 |
| 35-39 years | 1.123459355 | 0.502205051 | 0.009461994 | 0.004229115 |
| 40-44 years | 0.893620378 | 0.434808991 | 0.007529132 | 0.003662661 |
| 45-49 years | 0.678261332 | 0.351513953 | 0.005716253 | 0.002964178 |
| 50-54 years | 0.598863966 | 0.327092107 | 0.005047812 | 0.002758912 |
| 55-59 years | 0.61341367 | 0.337882888 | 0.005170131 | 0.00284821 |
| 60-64 years | 0.665190411 | 0.359277957 | 0.005602505 | 0.003026942 |
| 65-69 years | 0.724160254 | 0.380671989 | 0.006100223 | 0.003206866 |
| 70-74 years | 0.724395172 | 0.384946652 | 0.006103475 | 0.003241857 |
| 75-79 years | 0.745656647 | 0.390398012 | 0.006281993 | 0.003286999 |
| 80-84 years | 0.777403302 | 0.39471824 | 0.006549214 | 0.003322985 |
| 85-89 years | 0.85256002 | 0.420610322 | 0.007183338 | 0.003541058 |
| 90-94 years | 1.042115702 | 0.468019645 | 0.008780088 | 0.003940388 |
| 95+ years | 1.261858709 | 0.510107975 | 0.010629566 | 0.004294097 |
| **Index** | **Incidence Rate** | | | |
|  | **1990** | | **2023** | |
|  | **Male** | **Female** | **Male** | **Female** |
| **G20** | | | | |
| <5 years | 24.06567913 | 12.79420785 | 0.474475774 | 0.260743725 |
| 5-9 years | 24.11159306 | 9.465612783 | 0.380293982 | 0.15601388 |
| 10-14 years | 11.56195029 | 3.843714877 | 0.21492354 | 0.07710727 |
| 15-19 years | 5.896275611 | 1.857215725 | 0.138730429 | 0.048058377 |
| 20-24 years | 4.159435773 | 1.363497664 | 0.103709343 | 0.036991346 |
| 25-29 years | 4.316148263 | 1.560956556 | 0.104799275 | 0.039954032 |
| 30-34 years | 4.112967506 | 1.632841396 | 0.090738919 | 0.037469576 |
| 35-39 years | 3.457066606 | 1.504497938 | 0.075518992 | 0.035023457 |
| 40-44 years | 2.785384689 | 1.296838656 | 0.063893685 | 0.031931942 |
| 45-49 years | 2.227693792 | 1.040268938 | 0.045152003 | 0.023739634 |
| 50-54 years | 1.783305409 | 0.849670825 | 0.035920054 | 0.019861239 |
| 55-59 years | 1.729838279 | 0.831968805 | 0.034672419 | 0.019835932 |
| 60-64 years | 1.770376473 | 0.771966597 | 0.037411167 | 0.020435427 |
| 65-69 years | 1.786038985 | 0.717312734 | 0.037960614 | 0.020432275 |
| 70-74 years | 1.559567017 | 0.616157209 | 0.038170328 | 0.020571638 |
| 75-79 years | 1.466253885 | 0.480384171 | 0.039332817 | 0.019933045 |
| 80-84 years | 1.777787472 | 0.448205814 | 0.039670117 | 0.019377121 |
| 85-89 years | 1.655268902 | 0.366800624 | 0.040761845 | 0.018341741 |
| 90-94 years | 1.882939649 | 0.347503655 | 0.052118428 | 0.020080224 |
| 95+ years | 2.294810682 | 0.366976264 | 0.098319178 | 0.027994124 |
| **Brazil** | | | | |
| <5 years | 11.30505869 | 6.117045234 | 4.330384984 | 2.349378597 |
| 5-9 years | 10.67473953 | 4.294830573 | 3.73406424 | 1.49701312 |
| 10-14 years | 5.872802348 | 2.068822688 | 2.108087291 | 0.739531223 |
| 15-19 years | 3.626302699 | 1.234000022 | 1.354757999 | 0.461543793 |
| 20-24 years | 2.587151308 | 0.909267611 | 0.996762216 | 0.349234214 |
| 25-29 years | 2.662252477 | 0.984471632 | 1.06552623 | 0.394062679 |
| 30-34 years | 2.504315781 | 1.001905452 | 1.027982038 | 0.41032007 |
| 35-39 years | 2.085736868 | 0.934211196 | 0.863498934 | 0.386277604 |
| 40-44 years | 1.690060824 | 0.814945323 | 0.676104872 | 0.326411322 |
| 45-49 years | 1.318247685 | 0.671628539 | 0.510471674 | 0.261136112 |
| 50-54 years | 1.149525588 | 0.616878228 | 0.440394632 | 0.236876687 |
| 55-59 years | 1.165120504 | 0.621609719 | 0.445806942 | 0.242227613 |
| 60-64 years | 1.260616207 | 0.648732543 | 0.471612899 | 0.247576943 |
| 65-69 years | 1.421349771 | 0.697181165 | 0.508874202 | 0.261606458 |
| 70-74 years | 1.479640466 | 0.724946271 | 0.524496491 | 0.271032106 |
| 75-79 years | 1.552301964 | 0.74458753 | 0.553173803 | 0.269908156 |
| 80-84 years | 1.653796563 | 0.759080129 | 0.604904867 | 0.283957875 |
| 85-89 years | 1.845223331 | 0.785660465 | 0.675431874 | 0.303721699 |
| 90-94 years | 2.017056676 | 0.863946128 | 0.897157826 | 0.352398363 |
| 95+ years | 2.786944425 | 1.128941736 | 1.177369489 | 0.452510056 |
| **India** | | | | |
| <5 years | 71.13948907 | 38.09306131 | 0.524733365 | 0.283532931 |
| 5-9 years | 69.26060995 | 27.57951686 | 0.48293888 | 0.195618997 |
| 10-14 years | 35.67317574 | 12.08011161 | 0.269041923 | 0.095492937 |
| 15-19 years | 21.03992793 | 6.805822719 | 0.168103332 | 0.056796899 |
| 20-24 years | 15.83241669 | 5.325544475 | 0.11841875 | 0.040434766 |
| 25-29 years | 16.89437711 | 6.235723194 | 0.114843523 | 0.041029182 |
| 30-34 years | 16.47040244 | 6.640882461 | 0.102946299 | 0.039630589 |
| 35-39 years | 14.55646538 | 6.558887911 | 0.086674603 | 0.037953836 |
| 40-44 years | 11.60164836 | 5.602856951 | 0.069850328 | 0.032968056 |
| 45-49 years | 8.806057917 | 4.384054937 | 0.051473703 | 0.025137386 |
| 50-54 years | 7.812521924 | 4.012222295 | 0.042785605 | 0.021677448 |
| 55-59 years | 7.761776764 | 4.08065594 | 0.042611065 | 0.023944452 |
| 60-64 years | 8.384629703 | 4.202522921 | 0.047087442 | 0.023954014 |
| 65-69 years | 9.247969781 | 4.552914868 | 0.053775165 | 0.027496608 |
| 70-74 years | 8.580414648 | 4.184243698 | 0.060111967 | 0.031664293 |
| 75-79 years | 9.224629112 | 4.064146651 | 0.057805981 | 0.025441266 |
| 80-84 years | 10.9168677 | 4.114652168 | 0.046244143 | 0.021842896 |
| 85-89 years | 11.6619914 | 4.414353297 | 0.042388055 | 0.015362774 |
| 90-94 years | 14.12018351 | 5.119696039 | 0.051543425 | 0.013863419 |
| 95+ years | 15.79982832 | 5.734810069 | 0.066349607 | 0.015988584 |
| **Italy** | | | | |
| <5 years | 1.521951224 | 0.821147328 | 0.559420404 | 0.300663217 |
| 5-9 years | 1.473270017 | 0.588330733 | 0.532857248 | 0.213197955 |
| 10-14 years | 0.811907697 | 0.284552001 | 0.297280347 | 0.104466411 |
| 15-19 years | 0.493952966 | 0.168780509 | 0.192443067 | 0.065456231 |
| 20-24 years | 0.359396773 | 0.127102947 | 0.148925014 | 0.052015961 |
| 25-29 years | 0.381090169 | 0.14331601 | 0.162895422 | 0.060052858 |
| 30-34 years | 0.370010876 | 0.149972161 | 0.159230207 | 0.063282267 |
| 35-39 years | 0.30706707 | 0.139070967 | 0.133529793 | 0.059585091 |
| 40-44 years | 0.239822505 | 0.117434451 | 0.105675675 | 0.051653986 |
| 45-49 years | 0.174009504 | 0.091403582 | 0.078866546 | 0.041119933 |
| 50-54 years | 0.14958032 | 0.083482816 | 0.068042269 | 0.037852555 |
| 55-59 years | 0.156609324 | 0.087413294 | 0.07013588 | 0.039306651 |
| 60-64 years | 0.16964848 | 0.091257168 | 0.077327203 | 0.042353356 |
| 65-69 years | 0.183953373 | 0.094491429 | 0.085469263 | 0.045167637 |
| 70-74 years | 0.186504798 | 0.095745175 | 0.084191334 | 0.044726309 |
| 75-79 years | 0.18737792 | 0.091305027 | 0.084352099 | 0.044265322 |
| 80-84 years | 0.196218975 | 0.089332369 | 0.08412966 | 0.043053919 |
| 85-89 years | 0.221221834 | 0.093978062 | 0.092289692 | 0.045263508 |
| 90-94 years | 0.279930413 | 0.104817831 | 0.116662589 | 0.050064095 |
| 95+ years | 0.376132048 | 0.124490295 | 0.144429788 | 0.054270934 |
| **Turkey** | | | | |
| <5 years | 19.27385751 | 10.43559426 | 0.164898342 | 0.088670501 |
| 5-9 years | 18.52565863 | 7.42276433 | 0.156095271 | 0.062472775 |
| 10-14 years | 10.32808436 | 3.627755395 | 0.087004276 | 0.030529687 |
| 15-19 years | 6.553059039 | 2.230643665 | 0.055217069 | 0.018774572 |
| 20-24 years | 5.022361398 | 1.751582511 | 0.042296365 | 0.014748581 |
| 25-29 years | 5.522114807 | 2.029330258 | 0.046485382 | 0.017093721 |
| 30-34 years | 5.370935372 | 2.129772817 | 0.045236165 | 0.017943567 |
| 35-39 years | 4.49383742 | 2.008820203 | 0.037847976 | 0.01691646 |
| 40-44 years | 3.574481514 | 1.739235965 | 0.030116528 | 0.014650644 |
| 45-49 years | 2.713045326 | 1.406055814 | 0.022865012 | 0.01185671 |
| 50-54 years | 2.395455862 | 1.30836843 | 0.020191249 | 0.011035648 |
| 55-59 years | 2.453654681 | 1.351531551 | 0.020680524 | 0.011392839 |
| 60-64 years | 2.660761645 | 1.437111827 | 0.022410021 | 0.012107769 |
| 65-69 years | 2.896641017 | 1.522687957 | 0.024400892 | 0.012827462 |
| 70-74 years | 2.897580689 | 1.539786608 | 0.024413901 | 0.012967429 |
| 75-79 years | 2.98262659 | 1.56159205 | 0.025127973 | 0.013147996 |
| 80-84 years | 3.109613206 | 1.578872961 | 0.026196858 | 0.013291938 |
| 85-89 years | 3.41024008 | 1.682441286 | 0.028733354 | 0.014164233 |
| 90-94 years | 4.168462809 | 1.872078582 | 0.035120351 | 0.015761553 |
| 95+ years | 5.047434835 | 2.040431898 | 0.042518266 | 0.017176389 |
| **Index** | **Mortality rate** | | | |
|  | **1990** | | **2023** | |
|  | **Male** | **Female** | **Male** | **Female** |
| **G20** | | | | |
| <5 years | 3.280107444 | 2.516924919 | 0.170320684 | 0.109981879 |
| 5-9 years | 0.637597992 | 0.353317187 | 0.027354418 | 0.013725979 |
| 10-14 years | 0.304822963 | 0.150274889 | 0.023053627 | 0.010380958 |
| 15-19 years | 0.296686102 | 0.109495512 | 0.02797188 | 0.010697178 |
| 20-24 years | 0.268835417 | 0.104577975 | 0.027623365 | 0.012423746 |
| 25-29 years | 0.363726986 | 0.16410256 | 0.039556392 | 0.017460827 |
| 30-34 years | 0.449781553 | 0.196149846 | 0.040834317 | 0.019026458 |
| 35-39 years | 0.453411828 | 0.260035236 | 0.038692996 | 0.019675325 |
| 40-44 years | 0.502971899 | 0.295727127 | 0.039364074 | 0.020248121 |
| 45-49 years | 0.578301822 | 0.355832587 | 0.030778248 | 0.018230775 |
| 50-54 years | 0.549773898 | 0.299383975 | 0.025743279 | 0.014838006 |
| 55-59 years | 0.52644725 | 0.294826237 | 0.025624052 | 0.015895996 |
| 60-64 years | 0.593338909 | 0.307783176 | 0.029540527 | 0.017126943 |
| 65-69 years | 0.587786356 | 0.270315816 | 0.030725324 | 0.017497507 |
| 70-74 years | 0.476118715 | 0.206874151 | 0.031574619 | 0.017572192 |
| 75-79 years | 0.430519664 | 0.14924731 | 0.031655053 | 0.016567178 |
| 80-84 years | 0.476137891 | 0.13823235 | 0.030846147 | 0.015636029 |
| 85-89 years | 0.395771987 | 0.095343377 | 0.030224041 | 0.013972865 |
| 90-94 years | 0.439134953 | 0.091929053 | 0.037713251 | 0.014758083 |
| 95+ years | 0.487590067 | 0.092639224 | 0.071042794 | 0.02147344 |
| **Brazil** | | | | |
| <5 years | 2.799969713 | 1.648967906 | 2.050570342 | 1.248345948 |
| 5-9 years | 0.648038755 | 0.290821228 | 0.482139957 | 0.224992218 |
| 10-14 years | 0.40710866 | 0.15552332 | 0.355405078 | 0.144146929 |
| 15-19 years | 0.376019337 | 0.144540575 | 0.381970004 | 0.139920208 |
| 20-24 years | 0.387252883 | 0.151549083 | 0.385197004 | 0.146919596 |
| 25-29 years | 0.578355071 | 0.223513909 | 0.570566465 | 0.225432434 |
| 30-34 years | 0.671221009 | 0.266293735 | 0.636939888 | 0.26566424 |
| 35-39 years | 0.631847811 | 0.278655783 | 0.593859029 | 0.272400835 |
| 40-44 years | 0.547122132 | 0.25766909 | 0.499496986 | 0.249251912 |
| 45-49 years | 0.484481817 | 0.234837692 | 0.429995872 | 0.224888473 |
| 50-54 years | 0.433960883 | 0.226096959 | 0.381889384 | 0.211819588 |
| 55-59 years | 0.450704308 | 0.228105396 | 0.401796399 | 0.223392801 |
| 60-64 years | 0.513755644 | 0.255712765 | 0.449198586 | 0.238435936 |
| 65-69 years | 0.578370445 | 0.275910098 | 0.497398654 | 0.258194181 |
| 70-74 years | 0.621546881 | 0.305099828 | 0.515626907 | 0.268019964 |
| 75-79 years | 0.65052538 | 0.313910741 | 0.525409235 | 0.265632426 |
| 80-84 years | 0.761460393 | 0.348737136 | 0.577601567 | 0.276928233 |
| 85-89 years | 0.791610669 | 0.366378416 | 0.615305464 | 0.287052385 |
| 90-94 years | 0.933900131 | 0.463835126 | 0.773881029 | 0.318773997 |
| 95+ years | 0.995181348 | 0.607136688 | 0.962203391 | 0.399958639 |
| **India** | | | | |
| <5 years | 9.659240245 | 7.612933449 | 0.147597499 | 0.100648394 |
| 5-9 years | 1.801723953 | 1.029279464 | 0.016749325 | 0.00955233 |
| 10-14 years | 0.909025004 | 0.470053811 | 0.02004464 | 0.010293866 |
| 15-19 years | 1.048446768 | 0.397724459 | 0.0275959 | 0.010545809 |
| 20-24 years | 1.008783333 | 0.404567041 | 0.022670076 | 0.012299565 |
| 25-29 years | 1.395229121 | 0.649088554 | 0.032322468 | 0.015066211 |
| 30-34 years | 1.774791854 | 0.785088326 | 0.038556921 | 0.018465437 |
| 35-39 years | 1.888059313 | 1.136972432 | 0.03729385 | 0.019633696 |
| 40-44 years | 2.107161585 | 1.297864615 | 0.042862131 | 0.019694386 |
| 45-49 years | 2.317800234 | 1.540636911 | 0.034518627 | 0.022566957 |
| 50-54 years | 2.464945818 | 1.451469142 | 0.034806579 | 0.017962879 |
| 55-59 years | 2.405165533 | 1.483551184 | 0.035450647 | 0.023305483 |
| 60-64 years | 2.870563075 | 1.730244706 | 0.039107668 | 0.022631404 |
| 65-69 years | 3.101779873 | 1.765253422 | 0.046815977 | 0.02697136 |
| 70-74 years | 2.64393398 | 1.423268826 | 0.056167923 | 0.03134756 |
| 75-79 years | 2.74568499 | 1.28479664 | 0.054167945 | 0.025180076 |
| 80-84 years | 2.92134706 | 1.293950441 | 0.041645347 | 0.021574833 |
| 85-89 years | 2.766030501 | 1.149371061 | 0.040090992 | 0.015189381 |
| 90-94 years | 3.207656067 | 1.328540213 | 0.050888185 | 0.013723847 |
| 95+ years | 3.578733291 | 1.498508356 | 0.065686099 | 0.015828276 |
| **Italy** | | | | |
| <5 years | 0.128922495 | 0.072472257 | 0.050658803 | 0.026427782 |
| 5-9 years | 0.125593583 | 0.050852927 | 0.068811803 | 0.022159448 |
| 10-14 years | 0.070329489 | 0.025023563 | 0.036460281 | 0.014268257 |
| 15-19 years | 0.042417817 | 0.01474217 | 0.024118278 | 0.008547421 |
| 20-24 years | 0.031187366 | 0.011555099 | 0.018800119 | 0.006650783 |
| 25-29 years | 0.034742856 | 0.012963771 | 0.020770839 | 0.007350897 |
| 30-34 years | 0.032729573 | 0.01335124 | 0.020552887 | 0.008110772 |
| 35-39 years | 0.025684386 | 0.011777297 | 0.016997736 | 0.007313995 |
| 40-44 years | 0.019889259 | 0.00990158 | 0.0132399 | 0.006287274 |
| 45-49 years | 0.01413345 | 0.007566634 | 0.010090008 | 0.004959298 |
| 50-54 years | 0.011954541 | 0.0067973 | 0.008565911 | 0.004528661 |
| 55-59 years | 0.012739175 | 0.007145665 | 0.009063796 | 0.004837267 |
| 60-64 years | 0.013643222 | 0.007472576 | 0.009760491 | 0.005339929 |
| 65-69 years | 0.014888827 | 0.00775311 | 0.010819388 | 0.005732249 |
| 70-74 years | 0.015014531 | 0.007777044 | 0.01080378 | 0.005655062 |
| 75-79 years | 0.015209817 | 0.007487665 | 0.010607747 | 0.00567653 |
| 80-84 years | 0.015785815 | 0.007245197 | 0.010694782 | 0.005529797 |
| 85-89 years | 0.017737012 | 0.007430122 | 0.01160806 | 0.005788775 |
| 90-94 years | 0.021587718 | 0.007190433 | 0.015632956 | 0.006954111 |
| 95+ years | 0.031814348 | 0.00772868 | 0.017310616 | 0.006925348 |
| **Turkey** | | | | |
| <5 years | 0.443587488 | 0.250983515 | 0.002785821 | 0.001551414 |
| 5-9 years | 0.08765723 | 0.031930708 | 0.000375161 | 0.000166613 |
| 10-14 years | 0.057331431 | 0.019153966 | 0.000329381 | 0.000129469 |
| 15-19 years | 0.06991914 | 0.025111165 | 0.000403338 | 0.000168441 |
| 20-24 years | 0.073469367 | 0.024128679 | 0.000468398 | 0.000185628 |
| 25-29 years | 0.132304797 | 0.04573232 | 0.000782227 | 0.000297965 |
| 30-34 years | 0.157610605 | 0.061792356 | 0.000930793 | 0.000358843 |
| 35-39 years | 0.177879173 | 0.07885283 | 0.001024491 | 0.000439777 |
| 40-44 years | 0.172897538 | 0.086408069 | 0.000953012 | 0.00047911 |
| 45-49 years | 0.186477348 | 0.1066218 | 0.001039014 | 0.00060258 |
| 50-54 years | 0.172129286 | 0.106456434 | 0.001021982 | 0.000580131 |
| 55-59 years | 0.18868473 | 0.116882152 | 0.001245348 | 0.000736506 |
| 60-64 years | 0.242001067 | 0.134117509 | 0.001618323 | 0.000920385 |
| 65-69 years | 0.268926746 | 0.144585549 | 0.001935219 | 0.001073622 |
| 70-74 years | 0.300840169 | 0.154869976 | 0.002132926 | 0.001200238 |
| 75-79 years | 0.257271429 | 0.152969543 | 0.002353356 | 0.001296142 |
| 80-84 years | 0.283918554 | 0.168230287 | 0.002545861 | 0.001410594 |
| 85-89 years | 0.252738425 | 0.157831026 | 0.002350712 | 0.001302744 |
| 90-94 years | 0.314660361 | 0.187678576 | 0.002933132 | 0.00156331 |
| 95+ years | 0.33796361 | 0.205471437 | 0.003492222 | 0.001741944 |
| **Index** | **DALYs rate** | | | |
|  | **1990** | | **2023** | |
|  | **Male** | **Female** | **Male** | **Female** |
| **G20** | | | | |
| <5 years | 286.1641085 | 219.6369562 | 14.86281096 | 9.601080415 |
| 5-9 years | 53.3645725 | 29.52884052 | 2.270517825 | 1.139237345 |
| 10-14 years | 23.94325831 | 11.77674747 | 1.788049674 | 0.806270185 |
| 15-19 years | 21.65295516 | 7.981262658 | 2.026831685 | 0.776476857 |
| 20-24 years | 18.2756297 | 7.098546562 | 1.868466034 | 0.840294752 |
| 25-29 years | 22.89456567 | 10.33223505 | 2.482893703 | 1.095137322 |
| 30-34 years | 26.04731339 | 11.36875191 | 2.359311957 | 1.098210873 |
| 35-39 years | 23.96983395 | 13.74906661 | 2.042869452 | 1.038255293 |
| 40-44 years | 24.09566049 | 14.17907347 | 1.884850432 | 0.968980066 |
| 45-49 years | 24.88755127 | 15.32353296 | 1.324413811 | 0.783843025 |
| 50-54 years | 21.03799716 | 11.44983894 | 0.984052615 | 0.566638939 |
| 55-59 years | 17.66454801 | 9.883894228 | 0.859117318 | 0.532444184 |
| 60-64 years | 17.1555157 | 8.894317384 | 0.853692611 | 0.494640737 |
| 65-69 years | 14.37615997 | 6.605793859 | 0.747024366 | 0.425046385 |
| 70-74 years | 9.586595984 | 4.16558817 | 0.63199451 | 0.351467171 |
| 75-79 years | 6.913133324 | 2.397071844 | 0.507648198 | 0.26508825 |
| 80-84 years | 6.01739106 | 1.743527074 | 0.386329124 | 0.195092998 |
| 85-89 years | 3.989321303 | 0.958622692 | 0.300680139 | 0.138542522 |
| 90-94 years | 3.818464467 | 0.798527139 | 0.325807943 | 0.12743248 |
| 95+ years | 3.987364812 | 0.753119358 | 0.548021959 | 0.168933964 |
| **Brazil** | | | | |
| <5 years | 244.7238135 | 144.2479607 | 179.0405963 | 109.0420536 |
| 5-9 years | 53.9153703 | 24.21171937 | 39.96594546 | 18.65640803 |
| 10-14 years | 31.70686612 | 12.12073402 | 27.51685782 | 11.1843225 |
| 15-19 years | 27.31072858 | 10.51191485 | 27.61362327 | 10.14575499 |
| 20-24 years | 26.21610559 | 10.26381238 | 26.03023105 | 9.93092543 |
| 25-29 years | 36.31517195 | 14.03154254 | 35.81885786 | 14.13367218 |
| 30-34 years | 38.82629348 | 15.39159371 | 36.80968316 | 15.32961427 |
| 35-39 years | 33.41034503 | 14.72346373 | 31.35734267 | 14.36608502 |
| 40-44 years | 26.24020928 | 12.34744567 | 23.91741664 | 11.92398157 |
| 45-49 years | 20.86550403 | 10.10738328 | 18.50954049 | 9.67495287 |
| 50-54 years | 16.6215962 | 8.653935741 | 14.59751824 | 8.09177203 |
| 55-59 years | 15.13072906 | 7.653659321 | 13.47405953 | 7.485089894 |
| 60-64 years | 14.85741962 | 7.392004091 | 12.98383869 | 6.88496441 |
| 65-69 years | 14.11092157 | 6.727091081 | 12.10832404 | 6.276080771 |
| 70-74 years | 12.45291939 | 6.098605071 | 10.31923204 | 5.354820982 |
| 75-79 years | 10.43397247 | 5.024222095 | 8.418310428 | 4.244504001 |
| 80-84 years | 9.560296679 | 4.356929877 | 7.217711409 | 3.448438679 |
| 85-89 years | 7.932076504 | 3.657112817 | 6.112031479 | 2.840344714 |
| 90-94 years | 8.085151561 | 4.011744814 | 6.680930961 | 2.750480427 |
| 95+ years | 7.77902327 | 4.722113426 | 7.371367964 | 3.134709724 |
| **India** | | | | |
| <5 years | 842.5848193 | 664.2483535 | 12.86800336 | 8.778139227 |
| 5-9 years | 150.8325301 | 86.03044597 | 1.394893091 | 0.794321513 |
| 10-14 years | 71.45003894 | 36.84812591 | 1.55856888 | 0.800352836 |
| 15-19 years | 76.54265095 | 28.99472204 | 2.005542375 | 0.766380189 |
| 20-24 years | 68.59144847 | 27.46236389 | 1.535976441 | 0.832495418 |
| 25-29 years | 87.82137349 | 40.87071263 | 2.028305224 | 0.945607038 |
| 30-34 years | 102.7851364 | 45.51311326 | 2.226754771 | 1.066453756 |
| 35-39 years | 99.79364605 | 60.11017169 | 1.967724512 | 1.037006123 |
| 40-44 years | 100.9289302 | 62.22710149 | 2.052301114 | 0.943274834 |
| 45-49 years | 99.75001148 | 66.35033796 | 1.485686716 | 0.970071548 |
| 50-54 years | 94.31988379 | 55.50927558 | 1.330882758 | 0.685578723 |
| 55-59 years | 80.69990772 | 49.7300081 | 1.187477 | 0.779604323 |
| 60-64 years | 82.99432354 | 49.99689253 | 1.127795558 | 0.652861942 |
| 65-69 years | 75.87120779 | 43.1441225 | 1.135945526 | 0.65488927 |
| 70-74 years | 53.25158352 | 28.67481309 | 1.124777822 | 0.62869764 |
| 75-79 years | 44.08387375 | 20.63910489 | 0.870887473 | 0.404903552 |
| 80-84 years | 36.92868101 | 16.32916961 | 0.525765988 | 0.27084904 |
| 85-89 years | 27.9020883 | 11.56770539 | 0.400265908 | 0.151952505 |
| 90-94 years | 27.89914745 | 11.54268548 | 0.439727544 | 0.118598774 |
| 95+ years | 29.43483468 | 12.27932861 | 0.539213686 | 0.129860369 |
| **Italy** | | | | |
| <5 years | 11.29209242 | 6.358559946 | 4.431128713 | 2.316307664 |
| 5-9 years | 10.40842716 | 4.217214545 | 5.691525927 | 1.833172945 |
| 10-14 years | 5.452072834 | 1.943308486 | 2.827711973 | 1.106403578 |
| 15-19 years | 3.076649882 | 1.071943521 | 1.748727275 | 0.620985169 |
| 20-24 years | 2.114185288 | 0.783407106 | 1.272707408 | 0.450762021 |
| 25-29 years | 2.186924607 | 0.815233234 | 1.30446747 | 0.461205185 |
| 30-34 years | 1.898371931 | 0.772991004 | 1.188778567 | 0.468591791 |
| 35-39 years | 1.361956718 | 0.623352231 | 0.898276502 | 0.386247048 |
| 40-44 years | 0.955084904 | 0.475230087 | 0.633887035 | 0.300787064 |
| 45-49 years | 0.608746891 | 0.325839571 | 0.433925576 | 0.213187168 |
| 50-54 years | 0.458772857 | 0.260895466 | 0.327800836 | 0.173276995 |
| 55-59 years | 0.428050445 | 0.240196822 | 0.30435736 | 0.162379215 |
| 60-64 years | 0.395915507 | 0.216676235 | 0.282919298 | 0.154668299 |
| 65-69 years | 0.364615105 | 0.189313689 | 0.263907561 | 0.139811316 |
| 70-74 years | 0.302424256 | 0.156125998 | 0.215953704 | 0.112877156 |
| 75-79 years | 0.245559343 | 0.120321103 | 0.170932368 | 0.091050831 |
| 80-84 years | 0.201795455 | 0.091979251 | 0.133827385 | 0.068870862 |
| 85-89 years | 0.18017543 | 0.075258591 | 0.116047355 | 0.057556298 |
| 90-94 years | 0.191240792 | 0.063836987 | 0.136781605 | 0.060752127 |
| 95+ years | 0.268460784 | 0.065586374 | 0.144445946 | 0.057520086 |
| **Turkey** | | | | |
| <5 years | 39.11042563 | 22.14678085 | 0.245658735 | 0.136794859 |
| 5-9 years | 7.59634352 | 2.780962533 | 0.033767042 | 0.014872253 |
| 10-14 years | 4.638434641 | 1.553722506 | 0.027098868 | 0.010593635 |
| 15-19 years | 5.19137891 | 1.863014748 | 0.030216301 | 0.012567799 |
| 20-24 years | 5.05980568 | 1.664525515 | 0.032403758 | 0.012817238 |
| 25-29 years | 8.397480599 | 2.902997786 | 0.049902276 | 0.018994795 |
| 30-34 years | 9.189846545 | 3.605213079 | 0.054567938 | 0.021046213 |
| 35-39 years | 9.47144043 | 4.201265395 | 0.05472351 | 0.023498434 |
| 40-44 years | 8.34861934 | 4.163858263 | 0.0460721 | 0.023153332 |
| 45-49 years | 8.046567107 | 4.601261199 | 0.045041574 | 0.026108674 |
| 50-54 years | 6.618695145 | 4.085318732 | 0.039364432 | 0.022376305 |
| 55-59 years | 6.349366682 | 3.925798628 | 0.041916664 | 0.024774845 |
| 60-64 years | 7.037128456 | 3.895607072 | 0.047096317 | 0.026782132 |
| 65-69 years | 6.645961219 | 3.562235811 | 0.047372245 | 0.026211339 |
| 70-74 years | 6.050475606 | 3.109089842 | 0.043001916 | 0.024103729 |
| 75-79 years | 4.166808201 | 2.461991041 | 0.03805917 | 0.020889479 |
| 80-84 years | 3.619877422 | 2.126466844 | 0.032269364 | 0.01776922 |
| 85-89 years | 2.592469905 | 1.603931767 | 0.023781741 | 0.013143042 |
| 90-94 years | 2.78153507 | 1.650125882 | 0.025965988 | 0.013750873 |
| 95+ years | 2.841044814 | 1.695566856 | 0.029532105 | 0.0142777 |
