# Supplementary material for: Epidemiological transitions of Visceral Leishmaniasis in G20 countries: A 33-year retrospective joinpoint and demographic decomposition analysis with 20-year ARIMA projections
Source: PLoS Negl Trop Dis. 2026 Sep 16;20(9):e0014714. doi: 10.1371/journal.pntd.0014714 (PMC13580953; doi:10.1371/journal.pntd.0014714)
Supplement: Supplementary file 4 [file pntd.0014714.s004.docx]

**S4 Table** Trends in visceral leishmaniasis prevalence in G20 countries, decomposed by population aging, population growth and epidemiological changes, 1990-2023.

| **Location** | **Overall difference (cases)** | **Aging** | | **Population** | | **Epidemiological change** | |
| --- | --- | --- | --- | --- | --- | --- | --- |
|  |  | **Effect (cases)** | **Percent (%)** | **Effect (cases)** | **Percent (%)** | **Effect (cases)** | **Percent (%)** |
| G20 | 118214996 | 1063709.77 | 0.9 | 123664268 | 104.61 | -6512973.48 | -5.51 |
| Argentina | 1319519.84 | 31883.182 | 2.42 | 1344097.02 | 101.86 | -56467.364 | -4.28 |
| Brazil | 5279891.94 | -598364.296 | -11.33 | 5960758.42 | 112.9 | -82501.181 | -1.56 |
| China | 23012945.4 | -2666619.35 | -11.59 | 23867659.3 | 103.71 | 1811915.38 | 7.87 |
| European Union | 4225721.88 | -1589544.53 | -37.62 | 2708983.77 | 64.11 | 3106332.64 | 73.51 |
| France | 1344712.91 | -124168.475 | -9.23 | 1064908.34 | 79.19 | 403972.046 | 30.04 |
| India | 58116619.9 | -734918.784 | -1.26 | 58125878.5 | 100.02 | 724230.152 | 1.25 |
| Italy | 349134.68 | -231283.411 | -66.24 | 183541.418 | 52.57 | 396865.671 | 113.67 |
| Mexico | 4096505.98 | -12775.636 | -0.31 | 4448553.06 | 108.59 | -339271.445 | -8.28 |
| Saudi Arabia | 1657816.1 | 20958.766 | 1.26 | 1634250.13 | 98.58 | 2681.205 | 0.16 |
