# Supplementary material for: Epidemiological transitions of Visceral Leishmaniasis in G20 countries: A 33-year retrospective joinpoint and demographic decomposition analysis with 20-year ARIMA projections
Source: PLoS Negl Trop Dis. 2026 Sep 16;20(9):e0014714. doi: 10.1371/journal.pntd.0014714 (PMC13580953; doi:10.1371/journal.pntd.0014714)
Supplement: Supplementary file 5 [file pntd.0014714.s005.docx]

**S5 Table** Trends in visceral leishmaniasis incidence in G20 countries, decomposed by population aging, population growth and epidemiological changes, 1990-2023.

| **Location** | **Overall difference (cases)** | **Aging** | | **Population** | | **Epidemiological change** | |
| --- | --- | --- | --- | --- | --- | --- | --- |
|  |  | **Effect (cases)** | **Percent (%)** | **Effect (cases)** | **Percent (%)** | **Effect (cases)** | **Percent (%)** |
| G20 | 954002653.3 | -78947749.14 | -8.28 | 532587884.5 | 55.83 | 500362518.9 | 52.45 |
| Argentina | 6585918.57 | -1001482.567 | -15.21 | 6865109.842 | 104.24 | 722291.298 | 10.97 |
| Brazil | 56715310.2 | -306308.639 | -0.54 | 31984801.9 | 56.4 | 25036818.95 | 44.14 |
| China | 103116686 | -43071247.08 | -41.77 | 89850779.54 | 87.14 | 56337155.58 | 54.63 |
| European Union | 47829535.41 | -7577936.31 | -15.84 | 11646079.26 | 24.35 | 43761397.47 | 91.49 |
| France | 10271435.61 | -904865.18 | -8.81 | 4311727.587 | 41.98 | 6864574.206 | 66.83 |
| India | 239742922.8 | -9011051.029 | -3.76 | 256040478.9 | 106.8 | -7286505.019 | -3.04 |
| Italy | 2113009.18 | -1366676.712 | -64.68 | 809475.735 | 38.31 | 2670214.16 | 126.37 |
| Mexico | 21394095.59 | -3725678.039 | -17.41 | 22867958.15 | 106.89 | 2251818.48 | 10.53 |
| Saudi Arabia | 6613124.47 | -679337.454 | -10.27 | 7380662.919 | 111.61 | -88198.991 | -1.33 |
