# Supplementary material for: Epidemiological transitions of Visceral Leishmaniasis in G20 countries: A 33-year retrospective joinpoint and demographic decomposition analysis with 20-year ARIMA projections
Source: PLoS Negl Trop Dis. 2026 Sep 16;20(9):e0014714. doi: 10.1371/journal.pntd.0014714 (PMC13580953; doi:10.1371/journal.pntd.0014714)
Supplement: Supplementary file 6 [file pntd.0014714.s006.docx]

**S6 Table** Trends in visceral leishmaniasis mortality in G20 countries, decomposed by population aging, population growth and epidemiological changes, 1990-2023.

| **Location** | **Overall difference (cases)** | **Aging** | | **Population** | | **Epidemiological change** | |
| --- | --- | --- | --- | --- | --- | --- | --- |
|  |  | **Effect (cases)** | **Percent (%)** | **Effect (cases)** | **Percent (%)** | **Effect (cases)** | **Percent (%)** |
| G20 | 30818376.47 | 32347409.29 | 104.96 | 23758215.85 | 77.09 | -25287248.66 | -82.05 |
| Argentina | 134320.97 | 102817.87 | 76.55 | 131436.845 | 97.85 | -99933.748 | -74.4 |
| Brazil | 788750.27 | 730612.128 | 92.63 | 386726.916 | 49.03 | -328588.772 | -41.66 |
| China | 8790391.13 | 17278116.29 | 196.56 | 5024992.656 | 57.16 | -13512717.82 | -153.72 |
| European Union | 870451.51 | 2436847.324 | 279.95 | 539355.247 | 61.96 | -2105751.063 | -241.91 |
| France | 322639.62 | 274224.111 | 84.99 | 272615.54 | 84.5 | -224200.036 | -69.49 |
| India | 11252231.58 | 6988507.345 | 62.11 | 10216815.77 | 90.8 | -5953091.527 | -52.91 |
| Italy | 148095.33 | 384545.98 | 259.66 | 42405.749 | 28.63 | -278856.397 | -188.3 |
| Mexico | 222671.08 | 185672.013 | 83.38 | 205752.155 | 92.4 | -168753.091 | -75.79 |
| Saudi Arabia | -61822.98 | 49282.205 | 79.72 | 123851.638 | 200.33 | -234956.822 | -380.05 |
