# Supplementary material for: Epidemiological transitions of Visceral Leishmaniasis in G20 countries: A 33-year retrospective joinpoint and demographic decomposition analysis with 20-year ARIMA projections
Source: PLoS Negl Trop Dis. 2026 Sep 16;20(9):e0014714. doi: 10.1371/journal.pntd.0014714 (PMC13580953; doi:10.1371/journal.pntd.0014714)
Supplement: Supplementary file 7 [file pntd.0014714.s007.docx]

**S7 Table** Trends in visceral leishmaniasis DALYs in G20 countries, decomposed by population aging, population growth and epidemiological changes, 1990-2023.

| **Location** | **Overall difference (cases)** | **Aging** | | **Population** | | **Epidemiological change** | |
| --- | --- | --- | --- | --- | --- | --- | --- |
|  |  | **Effect (cases)** | **Percent (%)** | **Effect (cases)** | **Percent (%)** | **Effect (cases)** | **Percent (%)** |
| G20 | 552127878.8 | 280817481.4 | 50.86 | 561825060.6 | 101.76 | -290514663.2 | -52.62 |
| Argentina | 2769307.85 | 1391447.731 | 50.25 | 4483961.016 | 161.92 | -3106100.897 | -112.16 |
| Brazil | 26869261.39 | 18270132.18 | 68 | 17767171.52 | 66.12 | -9168042.306 | -34.12 |
| China | -177039179.1 | 213477881.9 | 120.58 | 99771556.06 | 56.36 | -490288617.1 | -276.94 |
| European Union | 7094064.04 | 30774035.97 | 433.8 | 14640474.47 | 206.38 | -38320446.39 | -540.18 |
| France | -4387860.24 | 2873885.597 | 65.5 | 4445088.043 | 101.3 | -11706833.88 | -266.8 |
| India | 108710581.8 | 29169277.6 | 26.83 | 327933043.1 | 301.66 | -248391738.9 | -228.49 |
| Italy | -6873454.52 | 3956323.53 | 57.56 | 950872.766 | 13.83 | -11780650.81 | -171.39 |
| Mexico | 25408193.81 | 12385934.05 | 48.75 | 18933365.96 | 74.52 | -5911106.203 | -23.26 |
| Saudi Arabia | 6393524.33 | 746168.034 | 11.67 | 5836090.42 | 91.28 | -188734.124 | -2.95 |
