# Supplementary material for: Epidemiological transitions of Visceral Leishmaniasis in G20 countries: A 33-year retrospective joinpoint and demographic decomposition analysis with 20-year ARIMA projections
Source: PLoS Negl Trop Dis. 2026 Sep 16;20(9):e0014714. doi: 10.1371/journal.pntd.0014714 (PMC13580953; doi:10.1371/journal.pntd.0014714)
Supplement: Supplementary file 8 [file pntd.0014714.s008.docx]

**S8 Table** Predicted all-age age-standardized prevalence rate, age-standardized incidence rate, age-standardized mortality rate, and age-standardized DALYs rate of visceral leishmaniasis in G20 from 2024 to 2043.

| **Year** | **Age-standardized prevalence rate**  **(per 100000)** | | | **Age-standardized incidence rate**  **(per 100000)** | | | **Age-standardized mortality rate**  **(per 100000)** | | | **Age-standardized DALYs rate**  **(per 100000)** | | |
| --- | --- | --- | --- | --- | --- | --- | --- | --- | --- | --- | --- | --- |
|  | **Both** | **Male** | **Female** | **Both** | **Male** | **Female** | **Both** | **Male** | **Female** | **Both** | **Male** | **Female** |
| 2024 | 0.030 | 0.040 | 0.018 | 0.119 | 0.162 | 0.071 | 0.035 | 0.046 | 0.024 | 2.362 | 3.061 | 1.617 |
| 2025 | 0.032 | 0.044 | 0.019 | 0.130 | 0.177 | 0.074 | 0.035 | 0.047 | 0.023 | 2.350 | 3.110 | 1.542 |
| 2026 | 0.037 | 0.050 | 0.020 | 0.146 | 0.199 | 0.080 | 0.035 | 0.048 | 0.021 | 2.338 | 3.158 | 1.466 |
| 2027 | 0.041 | 0.057 | 0.022 | 0.165 | 0.226 | 0.086 | 0.035 | 0.048 | 0.020 | 2.326 | 3.207 | 1.390 |
| 2028 | 0.047 | 0.064 | 0.024 | 0.187 | 0.257 | 0.093 | 0.035 | 0.049 | 0.019 | 2.314 | 3.255 | 1.315 |
| 2029 | 0.054 | 0.073 | 0.026 | 0.213 | 0.292 | 0.100 | 0.034 | 0.050 | 0.018 | 2.302 | 3.304 | 1.239 |
| 2030 | 0.060 | 0.083 | 0.028 | 0.240 | 0.330 | 0.107 | 0.034 | 0.051 | 0.017 | 2.289 | 3.353 | 1.163 |
| 2031 | 0.068 | 0.093 | 0.030 | 0.269 | 0.370 | 0.115 | 0.034 | 0.052 | 0.016 | 2.277 | 3.401 | 1.088 |
| 2032 | 0.076 | 0.104 | 0.032 | 0.300 | 0.413 | 0.123 | 0.034 | 0.053 | 0.015 | 2.265 | 3.450 | 1.012 |
| 2033 | 0.084 | 0.115 | 0.034 | 0.331 | 0.456 | 0.131 | 0.034 | 0.054 | 0.014 | 2.253 | 3.498 | 0.937 |
| 2034 | 0.092 | 0.126 | 0.036 | 0.364 | 0.501 | 0.139 | 0.034 | 0.055 | 0.013 | 2.241 | 3.547 | 0.861 |
| 2035 | 0.100 | 0.138 | 0.038 | 0.397 | 0.547 | 0.146 | 0.034 | 0.055 | 0.012 | 2.229 | 3.595 | 0.785 |
| 2036 | 0.109 | 0.149 | 0.041 | 0.430 | 0.594 | 0.154 | 0.034 | 0.056 | 0.010 | 2.217 | 3.644 | 0.710 |
| 2037 | 0.117 | 0.161 | 0.043 | 0.464 | 0.642 | 0.162 | 0.033 | 0.057 | 0.009 | 2.205 | 3.692 | 0.634 |
| 2038 | 0.126 | 0.173 | 0.045 | 0.499 | 0.690 | 0.170 | 0.033 | 0.058 | 0.008 | 2.193 | 3.741 | 0.558 |
| 2039 | 0.135 | 0.185 | 0.047 | 0.533 | 0.738 | 0.178 | 0.033 | 0.059 | 0.007 | 2.181 | 3.789 | 0.483 |
| 2040 | 0.144 | 0.198 | 0.049 | 0.568 | 0.786 | 0.186 | 0.033 | 0.060 | 0.006 | 2.169 | 3.838 | 0.407 |
| 2041 | 0.153 | 0.210 | 0.051 | 0.603 | 0.835 | 0.194 | 0.033 | 0.061 | 0.005 | 2.156 | 3.886 | 0.331 |
| 2042 | 0.162 | 0.222 | 0.053 | 0.639 | 0.884 | 0.202 | 0.033 | 0.062 | 0.004 | 2.144 | 3.935 | 0.256 |
| 2043 | 0.171 | 0.235 | 0.056 | 0.674 | 0.933 | 0.210 | 0.033 | 0.063 | 0.003 | 2.132 | 3.983 | 0.180 |
