# Supplementary material for: Epidemiological transitions of Visceral Leishmaniasis in G20 countries: A 33-year retrospective joinpoint and demographic decomposition analysis with 20-year ARIMA projections
Source: PLoS Negl Trop Dis. 2026 Sep 16;20(9):e0014714. doi: 10.1371/journal.pntd.0014714 (PMC13580953; doi:10.1371/journal.pntd.0014714)
Supplement: Supplementary file 9 [file pntd.0014714.s009.docx]

**S9 Table** Back-testing analysis of all-age age-standardized prevalence rate, age-standardized incidence rate, age-standardized mortality rate, and age-standardized DALYs rate burden prediction for visceral leishmaniasis in G20, 2014-2023.

| **Year** | **Age-standardized prevalence rate (per 100000)** | | | | | | | | |
| --- | --- | --- | --- | --- | --- | --- | --- | --- | --- |
|  | **Both** | | | **Male** | | | **Female** | | |
|  | **Actual** | **Forecast** | **MAE** | **Actual** | **Forecast** | **MAE** | **Actual** | **Forecast** | **MAE** |
| 2014 | 0.149 | 0.137 | 8.05 | 0.205 | 0.192 | 6.34 | 0.090 | 0.087 | 3.33 |
| 2015 | 0.117 | 0.114 | 2.56 | 0.161 | 0.157 | 2.48 | 0.071 | 0.078 | 9.86 |
| 2016 | 0.097 | 0.096 | 1.03 | 0.134 | 0.137 | 2.24 | 0.059 | 0.063 | 6.78 |
| 2017 | 0.080 | 0.078 | 2.50 | 0.109 | 0.112 | 2.75 | 0.048 | 0.054 | 12.5 |
| 2018 | 0.065 | 0.061 | 6.15 | 0.089 | 0.097 | 8.99 | 0.039 | 0.047 | 20.51 |
| 2019 | 0.053 | 0.049 | 7.55 | 0.072 | 0.074 | 2.78 | 0.032 | 0.042 | 31.25 |
| 2020 | 0.043 | 0.037 | 13.95 | 0.059 | 0.052 | 11.86 | 0.026 | 0.036 | 38.46 |
| 2021 | 0.036 | 0.031 | 13.89 | 0.048 | 0.047 | 2.08 | 0.022 | 0.029 | 31.82 |
| 2022 | 0.031 | 0.026 | 16.13 | 0.042 | 0.042 | 0 | 0.019 | 0.023 | 21.05 |
| 2023 | 0.029 | 0.021 | 27.59 | 0.039 | 0.037 | 5.13 | 0.018 | 0.017 | 5.56 |
| **MAPE** | - | - | **9.94%** | - | - | **4.47%** | - | - | **18.11%** |
| **Year** | **Age-standardized incidence rate (per 100000)** | | | | | | | | |
|  | **Both** | | | **Male** | | | **Female** | | |
|  | **Actual** | **Forecast** | **MAE** | **Actual** | **Forecast** | **MAE** | **Actual** | **Forecast** | **MAE** |
| 2014 | 0.598 | 0.581 | 2.84 | 0.820 | 0.797 | 2.80 | 0.359 | 0.371 | 3.34 |
| 2015 | 0.469 | 0.499 | 6.40 | 0.643 | 0.673 | 4.67 | 0.282 | 0.325 | 15.25 |
| 2016 | 0.390 | 0.422 | 8.21 | 0.534 | 0.557 | 4.31 | 0.235 | 0.283 | 20.43 |
| 2017 | 0.319 | 0.363 | 13.79 | 0.438 | 0.436 | 0.46 | 0.193 | 0.242 | 25.39 |
| 2018 | 0.259 | 0.298 | 15.06 | 0.355 | 0.321 | 9.58 | 0.157 | 0.214 | 36.31 |
| 2019 | 0.210 | 0.242 | 15.24 | 0.288 | 0.296 | 2.78 | 0.128 | 0.179 | 39.84 |
| 2020 | 0.173 | 0.194 | 12.14 | 0.236 | 0.218 | 7.63 | 0.105 | 0.142 | 35.24 |
| 2021 | 0.142 | 0.149 | 4.93 | 0.194 | 0.177 | 8.76 | 0.087 | 0.117 | 34.48 |
| 2022 | 0.123 | 0.103 | 16.26 | 0.167 | 0.149 | 10.78 | 0.075 | 0.085 | 13.33 |
| 2023 | 0.115 | 0.077 | 33.04 | 0.157 | 0.118 | 24.84 | 0.071 | 0.068 | 4.23 |
| **MAPE** | - | - | **12.79%** | - | - | **7.66%** | - | - | **22.78%** |
| **Year** | **Age-standardized mortality rate (per 100000)** | | | | | | | | |
|  | **Both** | | | **Male** | | | **Female** | | |
|  | **Actual** | **Forecast** | **MAE** | **Actual** | **Forecast** | **MAE** | **Actual** | **Forecast** | **MAE** |
| 2014 | 0.061 | 0.057 | 6.56 | 0.079 | 0.081 | 2.53 | 0.043 | 0.045 | 4.65 |
| 2015 | 0.052 | 0.050 | 3.85 | 0.067 | 0.074 | 10.45 | 0.036 | 0.041 | 13.89 |
| 2016 | 0.045 | 0.044 | 2.22 | 0.059 | 0.066 | 11.86 | 0.031 | 0.038 | 22.58 |
| 2017 | 0.042 | 0.039 | 7.14 | 0.055 | 0.059 | 7.27 | 0.029 | 0.033 | 13.79 |
| 2018 | 0.039 | 0.036 | 7.69 | 0.051 | 0.051 | 0 | 0.027 | 0.029 | 7.41 |
| 2019 | 0.037 | 0.032 | 13.51 | 0.048 | 0.044 | 8.33 | 0.026 | 0.025 | 3.85 |
| 2020 | 0.037 | 0.029 | 21.62 | 0.047 | 0.039 | 17.02 | 0.026 | 0.023 | 11.54 |
| 2021 | 0.036 | 0.025 | 30.56 | 0.047 | 0.034 | 27.66 | 0.025 | 0.021 | 16 |
| 2022 | 0.036 | 0.021 | 41.67 | 0.046 | 0.030 | 34.78 | 0.026 | 0.019 | 26.92 |
| 2023 | 0.035 | 0.018 | 48.57 | 0.045 | 0.028 | 37.78 | 0.025 | 0.016 | 36 |
| **MAPE** | - | - | **18.34%** | - | - | **15.77%** | - | - | **15.66%** |
| **Year** | **Age-standardized DALYs rate (per 100000)** | | | | | | | | |
|  | **Both** | | | **Male** | | | **Female** | | |
|  | **Actual** | **Forecast** | **MAE** | **Actual** | **Forecast** | **MAE** | **Actual** | **Forecast** | **MAE** |
| 2014 | 4.248 | 4.302 | 1.27 | 5.416 | 5.473 | 1.05 | 3.008 | 3.058 | 1.66 |
| 2015 | 3.543 | 3.927 | 10.84 | 4.530 | 4.938 | 9.01 | 2.494 | 2.751 | 10.31 |
| 2016 | 3.095 | 3.553 | 14.79 | 3.970 | 4.403 | 10.91 | 2.164 | 2.458 | 13.59 |
| 2017 | 2.861 | 3.288 | 14.92 | 3.675 | 3.968 | 7.97 | 1.995 | 2.186 | 9.57 |
| 2018 | 2.629 | 2.803 | 6.62 | 3.373 | 3.533 | 4.74 | 1.837 | 1.976 | 7.57 |
| 2019 | 2.493 | 2.657 | 6.58 | 3.182 | 3.102 | 2.51 | 1.759 | 1.814 | 3.13 |
| 2020 | 2.453 | 2.465 | 0.49 | 3.117 | 2.836 | 8.99 | 1.745 | 1.702 | 2.46 |
| 2021 | 2.461 | 2.321 | 5.69 | 3.138 | 2.671 | 14.88 | 1.740 | 1.569 | 9.83 |
| 2022 | 2.426 | 2.179 | 10.18 | 3.067 | 2.462 | 19.73 | 1.742 | 1.414 | 18.83 |
| 2023 | 2.374 | 1.978 | 16.68 | 3.013 | 2.241 | 25.62 | 1.693 | 1.242 | 26.64 |
| **MAPE** | - | - | **8.81%** | - | - | **10.54%** | - | - | **10.36%** |
