# Supplementary material for: Epidemiological transitions of Visceral Leishmaniasis in G20 countries: A 33-year retrospective joinpoint and demographic decomposition analysis with 20-year ARIMA projections
Source: PLoS Negl Trop Dis. 2026 Sep 16;20(9):e0014714. doi: 10.1371/journal.pntd.0014714 (PMC13580953; doi:10.1371/journal.pntd.0014714)
Supplement: Supplementary file 10 [file pntd.0014714.s010.docx]

**S10 Table** Predicted prevalence rate, incidence rate, mortality rate, and DALYs rate of visceral leishmaniasis aged <5 years in G20 from 2024 to 2043.

| **Year** | **Prevalence rate**  **(per 100000)** | | | **Incidence rate**  **(per 100000)** | | | **Mortality rate**  **(per 100000)** | | | **DALYs rate**  **(per 100000)** | | |
| --- | --- | --- | --- | --- | --- | --- | --- | --- | --- | --- | --- | --- |
|  | **Both** | **Male** | **Female** | **Both** | **Male** | **Female** | **Both** | **Male** | **Female** | **Both** | **Male** | **Female** |
| 2024 | 0.095 | 0.122 | 0.067 | 0.381 | 0.487 | 0.267 | 0.136 | 0.166 | 0.103 | 11.857 | 14.497 | 8.985 |
| 2025 | 0.102 | 0.130 | 0.071 | 0.407 | 0.521 | 0.285 | 0.130 | 0.162 | 0.096 | 11.375 | 14.132 | 8.369 |
| 2026 | 0.111 | 0.142 | 0.078 | 0.444 | 0.568 | 0.310 | 0.125 | 0.158 | 0.089 | 10.893 | 13.766 | 7.754 |
| 2027 | 0.122 | 0.156 | 0.085 | 0.487 | 0.625 | 0.340 | 0.119 | 0.154 | 0.082 | 10.411 | 13.401 | 7.138 |
| 2028 | 0.134 | 0.172 | 0.093 | 0.534 | 0.686 | 0.373 | 0.114 | 0.149 | 0.075 | 9.929 | 13.035 | 6.522 |
| 2029 | 0.146 | 0.188 | 0.102 | 0.584 | 0.751 | 0.408 | 0.108 | 0.145 | 0.068 | 9.447 | 12.670 | 5.906 |
| 2030 | 0.159 | 0.204 | 0.111 | 0.635 | 0.817 | 0.443 | 0.103 | 0.141 | 0.061 | 8.965 | 12.304 | 5.290 |
| 2031 | 0.172 | 0.221 | 0.120 | 0.686 | 0.884 | 0.479 | 0.097 | 0.137 | 0.054 | 8.483 | 11.939 | 4.674 |
| 2032 | 0.185 | 0.238 | 0.129 | 0.739 | 0.953 | 0.516 | 0.092 | 0.133 | 0.047 | 8.001 | 11.573 | 4.059 |
| 2033 | 0.198 | 0.255 | 0.138 | 0.791 | 1.021 | 0.552 | 0.086 | 0.128 | 0.040 | 7.519 | 11.208 | 3.443 |
| 2034 | 0.211 | 0.273 | 0.147 | 0.844 | 1.090 | 0.589 | 0.081 | 0.124 | 0.033 | 7.037 | 10.842 | 2.827 |
| 2035 | 0.224 | 0.290 | 0.156 | 0.897 | 1.159 | 0.626 | 0.075 | 0.120 | 0.026 | 6.554 | 10.477 | 2.211 |
| 2036 | 0.237 | 0.307 | 0.166 | 0.950 | 1.228 | 0.663 | 0.070 | 0.116 | 0.018 | 6.072 | 10.111 | 1.595 |
| 2037 | 0.251 | 0.324 | 0.175 | 1.003 | 1.297 | 0.700 | 0.064 | 0.112 | 0.011 | 5.590 | 9.746 | 0.979 |
| 2038 | 0.264 | 0.342 | 0.184 | 1.056 | 1.367 | 0.737 | 0.059 | 0.107 | 0.004 | 5.108 | 9.380 | 0.364 |
| 2039 | 0.277 | 0.359 | 0.193 | 1.109 | 1.436 | 0.774 | 0.053 | 0.103 | -0.003 | 4.626 | 9.015 | -0.252 |
| 2040 | 0.290 | 0.376 | 0.203 | 1.162 | 1.505 | 0.811 | 0.047 | 0.099 | -0.010 | 4.144 | 8.649 | -0.868 |
| 2041 | 0.304 | 0.394 | 0.212 | 1.215 | 1.574 | 0.848 | 0.042 | 0.095 | -0.017 | 3.662 | 8.284 | -1.484 |
| 2042 | 0.317 | 0.411 | 0.221 | 1.268 | 1.643 | 0.885 | 0.036 | 0.091 | -0.024 | 3.180 | 7.918 | -2.100 |
| 2043 | 0.330 | 0.428 | 0.230 | 1.321 | 1.713 | 0.922 | 0.031 | 0.086 | -0.031 | 2.698 | 7.553 | -2.716 |
