# Supplementary material for: Epidemiological transitions of Visceral Leishmaniasis in G20 countries: A 33-year retrospective joinpoint and demographic decomposition analysis with 20-year ARIMA projections
Source: PLoS Negl Trop Dis. 2026 Sep 16;20(9):e0014714. doi: 10.1371/journal.pntd.0014714 (PMC13580953; doi:10.1371/journal.pntd.0014714)
Supplement: Supplementary file 11 [file pntd.0014714.s011.docx]

**S11 Table** Predicted prevalence rate, incidence rate, mortality rate, and DALYs rate of visceral leishmaniasis aged ≥95 years in G20 from 2024 to 2043.

| **Year** | **Prevalence rate**  **(per 100000)** | | | **Incidence rate**  **(per 100000)** | | | **Mortality rate**  **(per 100000)** | | | **DALYs rate**  **(per 100000)** | | |
| --- | --- | --- | --- | --- | --- | --- | --- | --- | --- | --- | --- | --- |
|  | **Both** | **Male** | **Female** | **Both** | **Male** | **Female** | **Both** | **Male** | **Female** | **Both** | **Male** | **Female** |
| 2024 | 0.009 | 0.019 | 0.005 | 0.042 | 0.083 | 0.026 | 0.035 | 0.071 | 0.021 | 0.268 | 0.538 | 0.162 |
| 2025 | 0.008 | 0.017 | 0.005 | 0.038 | 0.078 | 0.024 | 0.035 | 0.071 | 0.020 | 0.264 | 0.531 | 0.157 |
| 2026 | 0.007 | 0.015 | 0.004 | 0.034 | 0.073 | 0.022 | 0.035 | 0.071 | 0.020 | 0.263 | 0.525 | 0.152 |
| 2027 | 0.006 | 0.013 | 0.004 | 0.030 | 0.068 | 0.021 | 0.035 | 0.071 | 0.019 | 0.262 | 0.521 | 0.147 |
| 2028 | 0.006 | 0.011 | 0.004 | 0.026 | 0.063 | 0.019 | 0.035 | 0.071 | 0.019 | 0.261 | 0.517 | 0.143 |
| 2029 | 0.005 | 0.009 | 0.003 | 0.022 | 0.058 | 0.017 | 0.035 | 0.071 | 0.019 | 0.261 | 0.514 | 0.140 |
| 2030 | 0.004 | 0.007 | 0.003 | 0.018 | 0.053 | 0.016 | 0.035 | 0.071 | 0.019 | 0.261 | 0.512 | 0.137 |
| 2031 | 0.003 | 0.005 | 0.002 | 0.014 | 0.048 | 0.014 | 0.035 | 0.071 | 0.018 | 0.261 | 0.511 | 0.134 |
| 2032 | 0.003 | 0.003 | 0.002 | 0.010 | 0.043 | 0.012 | 0.035 | 0.071 | 0.018 | 0.260 | 0.509 | 0.132 |
| 2033 | 0.002 | 0.001 | 0.002 | 0.006 | 0.038 | 0.011 | 0.035 | 0.071 | 0.018 | 0.260 | 0.508 | 0.130 |
| 2034 | 0.001 | -0.001 | 0.001 | 0.002 | 0.033 | 0.009 | 0.035 | 0.071 | 0.018 | 0.260 | 0.507 | 0.128 |
| 2035 | 0.000 | -0.003 | 0.001 | -0.002 | 0.028 | 0.007 | 0.035 | 0.071 | 0.018 | 0.260 | 0.507 | 0.127 |
| 2036 | 0.000 | -0.005 | 0.000 | -0.006 | 0.023 | 0.006 | 0.035 | 0.071 | 0.017 | 0.260 | 0.506 | 0.125 |
| 2037 | -0.001 | -0.007 | 0.000 | -0.010 | 0.018 | 0.004 | 0.035 | 0.071 | 0.017 | 0.260 | 0.506 | 0.124 |
| 2038 | -0.002 | -0.009 | -0.001 | -0.014 | 0.013 | 0.002 | 0.035 | 0.071 | 0.017 | 0.260 | 0.506 | 0.123 |
| 2039 | -0.003 | -0.011 | -0.001 | -0.018 | 0.008 | 0.001 | 0.035 | 0.071 | 0.017 | 0.260 | 0.506 | 0.122 |
| 2040 | -0.003 | -0.013 | -0.001 | -0.022 | 0.003 | -0.001 | 0.035 | 0.071 | 0.017 | 0.260 | 0.505 | 0.121 |
| 2041 | -0.004 | -0.015 | -0.002 | -0.026 | -0.002 | -0.001 | 0.035 | 0.071 | 0.017 | 0.260 | 0.505 | 0.120 |
| 2042 | -0.005 | -0.017 | -0.002 | -0.030 | -0.007 | -0.003 | 0.035 | 0.071 | 0.017 | 0.260 | 0.505 | 0.120 |
| 2043 | -0.005 | -0.019 | -0.003 | -0.034 | -0.012 | -0.005 | 0.035 | 0.071 | 0.017 | 0.260 | 0.505 | 0.119 |
