# Supplementary material for: Epidemiological transitions of Visceral Leishmaniasis in G20 countries: A 33-year retrospective joinpoint and demographic decomposition analysis with 20-year ARIMA projections
Source: PLoS Negl Trop Dis. 2026 Sep 16;20(9):e0014714. doi: 10.1371/journal.pntd.0014714 (PMC13580953; doi:10.1371/journal.pntd.0014714)
Supplement: Supplementary file 12 [file pntd.0014714.s012.docx]

**S12 Table** Back-testing analysis of prevalence rate, incidence rate, mortality rate, and DALYs rate burden prediction for visceral leishmaniasis aged <5 years in G20, 2014-2023.

| **Year** | **Prevalence rate (per 100000)** | | | | | | | | |
| --- | --- | --- | --- | --- | --- | --- | --- | --- | --- |
|  | **Both** | | | **Male** | | | **Female** | | |
|  | **Actual** | **Forecast** | **MAE** | **Actual** | **Forecast** | **MAE** | **Actual** | **Forecast** | **MAE** |
| 2014 | 0.461 | 0.467 | 1.3 | 0.588 | 0.582 | 1.02 | 0.323 | 0.328 | 1.55 |
| 2015 | 0.357 | 0.409 | 14.57 | 0.454 | 0.497 | 9.47 | 0.250 | 0.280 | 12 |
| 2016 | 0.292 | 0.351 | 20.21 | 0.372 | 0.412 | 10.75 | 0.205 | 0.232 | 13.17 |
| 2017 | 0.238 | 0.293 | 23.11 | 0.303 | 0.327 | 7.92 | 0.167 | 0.184 | 10.18 |
| 2018 | 0.194 | 0.235 | 21.13 | 0.247 | 0.242 | 2.02 | 0.136 | 0.136 | 0 |
| 2019 | 0.159 | 0.177 | 11.32 | 0.202 | 0.196 | 2.97 | 0.111 | 0.098 | 11.71 |
| 2020 | 0.132 | 0.119 | 9.85 | 0.168 | 0.152 | 9.52 | 0.093 | 0.084 | 9.68 |
| 2021 | 0.111 | 0.061 | 45.05 | 0.141 | 0.104 | 26.24 | 0.078 | 0.076 | 2.56 |
| 2022 | 0.097 | 0.003 | 96.91 | 0.124 | 0.058 | 53.23 | 0.068 | 0.068 | 0 |
| 2023 | 0.093 | -0.055 | 159.14 | 0.119 | 0.016 | 86.55 | 0.065 | 0.054 | 16.92 |
| **MAPE** | - | - | **40.26%** | - | - | **20.97%** | - | - | **7.78%** |
| **Year** | **Incidence rate (per 100000)** | | | | | | | | |
|  | **Both** | | | **Male** | | | **Female** | | |
|  | **Actual** | **Forecast** | **MAE** | **Actual** | **Forecast** | **MAE** | **Actual** | **Forecast** | **MAE** |
| 2014 | 1.844 | 1.988 | 7.8 | 2.351 | 2.379 | 1.19 | 1.292 | 1.314 | 1.7 |
| 2015 | 1.426 | 1.574 | 10.38 | 1.817 | 1.898 | 4.46 | 1.000 | 1.197 | 19.7 |
| 2016 | 1.170 | 1.267 | 8.29 | 1.490 | 1.439 | 3.42 | 0.820 | 1.080 | 31.71 |
| 2017 | 0.953 | 0.954 | 0.1 | 1.214 | 1.087 | 10.46 | 0.669 | 0.963 | 43.95 |
| 2018 | 0.776 | 0.679 | 12.5 | 0.988 | 0.874 | 11.54 | 0.545 | 0.846 | 55.23 |
| 2019 | 0.635 | 0.465 | 26.77 | 0.809 | 0.751 | 7.17 | 0.446 | 0.729 | 63.45 |
| 2020 | 0.529 | 0.275 | 48.02 | 0.674 | 0.628 | 6.82 | 0.371 | 0.612 | 64.96 |
| 2021 | 0.444 | 0.151 | 65.99 | 0.566 | 0.505 | 10.78 | 0.312 | 0.495 | 58.65 |
| 2022 | 0.389 | 0.053 | 86.38 | 0.496 | 0.382 | 22.98 | 0.273 | 0.378 | 38.46 |
| 2023 | 0.372 | -0.027 | 107.26 | 0.474 | 0.159 | 66.46 | 0.261 | 0.261 | 0 |
| **MAPE** | **-** | **-** | **37.35%** | **-** | **-** | **14.53%** | **-** | **-** | **37.78%** |
| **Year** | **Mortality rate (per 100000)** | | | | | | | | |
|  | **Both** | | | **Male** | | | **Female** | | |
|  | **Actual** | **Forecast** | **MAE** | **Actual** | **Forecast** | **MAE** | **Actual** | **Forecast** | **MAE** |
| 2014 | 0.262 | 0.267 | 1.91 | 0.312 | 0.314 | 0.64 | 0.208 | 0.216 | 3.85 |
| 2015 | 0.214 | 0.236 | 10.28 | 0.257 | 0.271 | 5.45 | 0.167 | 0.183 | 9.58 |
| 2016 | 0.181 | 0.205 | 13.26 | 0.219 | 0.227 | 3.65 | 0.140 | 0.150 | 7.14 |
| 2017 | 0.163 | 0.174 | 6.75 | 0.198 | 0.183 | 7.58 | 0.125 | 0.117 | 6.4 |
| 2018 | 0.149 | 0.143 | 4.03 | 0.181 | 0.139 | 23.2 | 0.115 | 0.104 | 9.57 |
| 2019 | 0.141 | 0.112 | 20.57 | 0.170 | 0.117 | 31.18 | 0.109 | 0.091 | 16.51 |
| 2020 | 0.141 | 0.081 | 42.55 | 0.169 | 0.095 | 43.79 | 0.111 | 0.078 | 29.73 |
| 2021 | 0.144 | 0.050 | 65.28 | 0.174 | 0.073 | 58.05 | 0.112 | 0.065 | 41.96 |
| 2022 | 0.145 | 0.019 | 86.9 | 0.174 | 0.051 | 70.69 | 0.113 | 0.052 | 53.98 |
| 2023 | 0.141 | 0.012 | 91.49 | 0.170 | 0.029 | 82.94 | 0.11 | 0.039 | 64.55 |
| **MAPE** | - | - | **34.30%** | - | - | **32.72%** | - | - | **24.33%** |
| **Year** | **DALYs rate (per 100000)** | | | | | | | | |
|  | **Both** | | | **Male** | | | **Female** | | |
|  | **Actual** | **Forecast** | **MAE** | **Actual** | **Forecast** | **MAE** | **Actual** | **Forecast** | **MAE** |
| 2014 | 22.882 | 23.313 | 1.88 | 27.205 | 27.422 | 0.8 | 18.172 | 18.85 | 3.73 |
| 2015 | 18.671 | 20.193 | 8.15 | 22.415 | 24.307 | 8.44 | 14.589 | 15.627 | 7.12 |
| 2016 | 15.812 | 17.073 | 7.97 | 19.116 | 21.192 | 10.86 | 12.206 | 12.404 | 1.62 |
| 2017 | 14.249 | 13.953 | 2.08 | 17.267 | 18.077 | 4.69 | 10.953 | 11.181 | 2.08 |
| 2018 | 13.052 | 12.823 | 1.75 | 15.791 | 16.962 | 7.42 | 10.058 | 9.958 | 0.99 |
| 2019 | 12.327 | 11.693 | 5.14 | 14.856 | 15.847 | 6.67 | 9.564 | 8.735 | 8.67 |
| 2020 | 12.322 | 10.563 | 14.28 | 14.768 | 14.732 | 0.24 | 9.653 | 8.012 | 17 |
| 2021 | 12.581 | 9.433 | 24.94 | 15.155 | 13.617 | 10.15 | 9.777 | 7.289 | 25.45 |
| 2022 | 12.663 | 8.303 | 34.43 | 15.199 | 12.502 | 17.75 | 9.906 | 6.566 | 33.72 |
| 2023 | 12.339 | 7.173 | 41.87 | 14.863 | 11.387 | 23.39 | 9.601 | 5.843 | 39.14 |
| **MAPE** | - | - | **14.26%** | - | - | **9.04%** | - | - | **13.95%** |
