# Supplementary material for: Epidemiological transitions of Visceral Leishmaniasis in G20 countries: A 33-year retrospective joinpoint and demographic decomposition analysis with 20-year ARIMA projections
Source: PLoS Negl Trop Dis. 2026 Sep 16;20(9):e0014714. doi: 10.1371/journal.pntd.0014714 (PMC13580953; doi:10.1371/journal.pntd.0014714)
Supplement: Supplementary file 13 [file pntd.0014714.s013.docx]

**S13 Table** Back-testing analysis of age-standardized prevalence rate, age-standardized incidence rate, age-standardized mortality rate, and age-standardized DALYs rate burden prediction for visceral leishmaniasis aged ≥95 years in G20, 2014-2023.

| **Year** | **Prevalence rate (per 100000)** | | | | | | | | |
| --- | --- | --- | --- | --- | --- | --- | --- | --- | --- |
|  | **Both** | | | **Male** | | | **Female** | | |
|  | **Actual** | **Forecast** | **MAE** | **Actual** | **Forecast** | **MAE** | **Actual** | **Forecast** | **MAE** |
| 2014 | 0.038 | 0.036 | 5.263 | 0.095 | 0.095 | 0 | 0.018 | 0.018 | 0 |
| 2015 | 0.033 | 0.032 | 3.030 | 0.08 | 0.087 | 8.75 | 0.016 | 0.017 | 4.17 |
| 2016 | 0.028 | 0.029 | 3.571 | 0.067 | 0.079 | 17.91 | 0.014 | 0.015 | 9.52 |
| 2017 | 0.025 | 0.026 | 4 | 0.058 | 0.071 | 22.41 | 0.013 | 0.014 | 7.69 |
| 2018 | 0.021 | 0.023 | 9.524 | 0.05 | 0.063 | 26 | 0.011 | 0.013 | 15.15 |
| 2019 | 0.019 | 0.020 | 5.263 | 0.043 | 0.055 | 27.91 | 0.01 | 0.011 | 13.33 |
| 2020 | 0.016 | 0.017 | 6.250 | 0.034 | 0.047 | 38.24 | 0.009 | 0.010 | 11.11 |
| 2021 | 0.014 | 0.014 | 0 | 0.032 | 0.039 | 21.88 | 0.008 | 0.009 | 8.33 |
| 2022 | 0.013 | 0.011 | 15.385 | 0.027 | 0.031 | 14.81 | 0.007 | 0.007 | 4.76 |
| 2023 | 0.011 | 0.008 | 27.273 | 0.023 | 0.023 | 0 | 0.006 | 0.006 | 0 |
| **MAPE** | - | - | **7.96%** | - | - | **17.79%** | - | - | **7.41%** |
| **Year** | **Incidence rate (per 100000)** | | | | | | | | |
|  | **Both** | | | **Male** | | | **Female** | | |
|  | **Actual** | **Forecast** | **MAE** | **Actual** | **Forecast** | **MAE** | **Actual** | **Forecast** | **MAE** |
| 2014 | 0.156 | 0.158 | 1.28 | 0.387 | 0.391 | 1.03 | 0.076 | 0.077 | 1.32 |
| 2015 | 0.134 | 0.146 | 8.62 | 0.325 | 0.357 | 9.98 | 0.068 | 0.072 | 5.23 |
| 2016 | 0.116 | 0.133 | 14.75 | 0.275 | 0.324 | 17.78 | 0.059 | 0.066 | 12.05 |
| 2017 | 0.102 | 0.121 | 18.3 | 0.240 | 0.290 | 20.97 | 0.053 | 0.061 | 14.47 |
| 2018 | 0.088 | 0.108 | 22.98 | 0.206 | 0.257 | 24.65 | 0.046 | 0.055 | 20.05 |
| 2019 | 0.078 | 0.096 | 22.79 | 0.179 | 0.223 | 24.71 | 0.041 | 0.050 | 21.41 |
| 2020 | 0.066 | 0.083 | 26.26 | 0.148 | 0.190 | 28.15 | 0.036 | 0.044 | 23.15 |
| 2021 | 0.060 | 0.071 | 18.15 | 0.131 | 0.156 | 19.17 | 0.033 | 0.039 | 17.85 |
| 2022 | 0.053 | 0.058 | 10.27 | 0.114 | 0.123 | 7.5 | 0.030 | 0.033 | 11.48 |
| 2023 | 0.048 | 0.046 | 4.17 | 0.098 | 0.089 | 9.180 | 0.028 | 0.028 | 0 |
| **MAPE** | - | - | **14.76%** | - | - | **16.31%** | - | - | **12.70%** |
| **Year** | **Mortality rate (per 100000)** | | | | | | | | |
|  | **Both** | | | **Male** | | | **Female** | | |
|  | **Actual** | **Forecast** | **MAE** | **Actual** | **Forecast** | **MAE** | **Actual** | **Forecast** | **MAE** |
| 2014 | 0.070 | 0.073 | 4.29 | 0.169 | 0.173 | 2.37 | 0.035 | 0.037 | 5.71 |
| 2015 | 0.061 | 0.069 | 12.93 | 0.146 | 0.162 | 10.65 | 0.032 | 0.035 | 9.72 |
| 2016 | 0.056 | 0.065 | 15.68 | 0.130 | 0.150 | 15.47 | 0.029 | 0.033 | 14.56 |
| 2017 | 0.053 | 0.061 | 14.47 | 0.123 | 0.139 | 12.74 | 0.028 | 0.031 | 11.9 |
| 2018 | 0.049 | 0.057 | 15.42 | 0.113 | 0.127 | 12.59 | 0.027 | 0.029 | 9.05 |
| 2019 | 0.047 | 0.052 | 11.58 | 0.105 | 0.116 | 10.26 | 0.026 | 0.028 | 6 |
| 2020 | 0.045 | 0.048 | 7.41 | 0.098 | 0.104 | 6.46 | 0.025 | 0.026 | 2.67 |
| 2021 | 0.041 | 0.044 | 7.86 | 0.087 | 0.093 | 6.77 | 0.024 | 0.024 | 0.93 |
| 2022 | 0.037 | 0.040 | 8.41 | 0.076 | 0.081 | 7.16 | 0.022 | 0.022 | 0.5 |
| 2023 | 0.035 | 0.036 | 2.86 | 0.071 | 0.070 | 1.41 | 0.021 | 0.020 | 4.76 |
| **MAPE** | - | - | **10.09%** | - | - | **8.59%** | - | - | **7.18%** |
| **Year** | **DALYs rate (per 100000)** | | | | | | | | |
|  | **Both** | | | **Male** | | | **Female** | | |
|  | **Actual** | **Forecast** | **MAE** | **Actual** | **Forecast** | **MAE** | **Actual** | **Forecast** | **MAE** |
| 2014 | 0.554 | 0.558 | 0.72 | 1.338 | 1.341 | 0.22 | 0.282 | 0.311 | 10.28 |
| 2015 | 0.484 | 0.526 | 8.59 | 1.155 | 1.252 | 8.43 | 0.252 | 0.296 | 17.33 |
| 2016 | 0.440 | 0.493 | 12.07 | 1.024 | 1.164 | 13.64 | 0.233 | 0.280 | 20.31 |
| 2017 | 0.415 | 0.461 | 11 | 0.960 | 1.075 | 11.98 | 0.222 | 0.265 | 19.37 |
| 2018 | 0.388 | 0.428 | 10.37 | 0.881 | 0.986 | 11.96 | 0.212 | 0.250 | 17.77 |
| 2019 | 0.371 | 0.396 | 6.68 | 0.821 | 0.898 | 9.34 | 0.207 | 0.234 | 13.20 |
| 2020 | 0.351 | 0.363 | 3.51 | 0.765 | 0.809 | 5.75 | 0.198 | 0.219 | 10.61 |
| 2021 | 0.326 | 0.331 | 1.5 | 0.685 | 0.720 | 5.16 | 0.192 | 0.204 | 6.08 |
| 2022 | 0.290 | 0.298 | 2.91 | 0.591 | 0.632 | 6.88 | 0.176 | 0.188 | 7.01 |
| 2023 | 0.275 | 0.266 | 3.27 | 0.548 | 0.543 | 0.91 | 0.169 | 0.173 | 2.37 |
| **MAPE** | - | - | **6.06%** | - | - | **7.43%** | - | - | **12.43%** |
