# Supplementary material for: Epidemiological transitions of Visceral Leishmaniasis in G20 countries: A 33-year retrospective joinpoint and demographic decomposition analysis with 20-year ARIMA projections
Source: PLoS Negl Trop Dis. 2026 Sep 16;20(9):e0014714. doi: 10.1371/journal.pntd.0014714 (PMC13580953; doi:10.1371/journal.pntd.0014714)
Supplement: Supplementary file 14 [file pntd.0014714.s014.docx]

**S14 Table** Optimal Parameters of Gender-Stratified ARIMA Models Selected by the auto.arima() Function.

| Indicator | Sex | Age | ARIMA(p,d,q) | AIC |
| --- | --- | --- | --- | --- |
| ASPR | Both | All-age | (3, 2, 0) | -212.57 |
| ASPR | Male | All-age | (3, 2, 0) | -191.8 |
| ASPR | Female | All-age | (2, 2, 1) | -253.1 |
| ASIR | Both | All-age | (3, 2, 0) | -123.84 |
| ASIR | Male | All-age | (3, 2, 0) | -103.08 |
| ASIR | Female | All-age | (2, 2, 1) | -253.1 |
| ASMR | Both | All-age | (0, 2, 1) | -215.67 |
| ASMR | Male | All-age | (0, 2, 1) | -206.17 |
| ASMR | Female | All-age | (0, 2, 1) | -226.43 |
| ASDR | Both | All-age | (0, 2, 1) | 57.96 |
| ASDR | Male | All-age | (0, 2, 1) | 65.94 |
| ASDR | Female | All-age | (0, 2, 1) | 49.18 |
| ASPR | Both | <5 years | (2, 2, 1) | -141.43 |
| ASPR | Male | <5 years | (2, 2, 1) | -125.3 |
| ASPR | Female | <5 years | (2, 2, 1) | -165.5 |
| ASIR | Both | <5 years | (2, 2, 1) | -52.71 |
| ASIR | Male | <5 years | (2, 2, 1) | -36.58 |
| ASIR | Female | <5 years | (2, 2, 1) | -76.78 |
| ASMR | Both | <5 years | (0, 2, 1) | -112.32 |
| ASMR | Male | <5 years | (0, 2, 1) | -111.26 |
| ASMR | Female | <5 years | (0, 2, 1) | -112.24 |
| ASDR | Both | <5 years | (0, 2, 1) | 173.66 |
| ASDR | Male | <5 years | (0, 2, 1) | 174.74 |
| ASDR | Female | <5 years | (0, 2, 1) | 173.73 |
| ASPR | Both | ≥95 years | (2, 2, 0) | -279.19 |
| ASPR | Male | ≥95 years | (1, 2, 0) | -218.99 |
| ASPR | Female | ≥95 years | (2, 2, 0) | -332.16 |
| ASIR | Both | ≥95 years | (2, 2, 0) | -192.01 |
| ASIR | Male | ≥95 years | (1, 2, 0) | -132.2 |
| ASIR | Female | ≥95 years | (0, 2, 2) | -244.48 |
| ASMR | Both | ≥95 years | (2, 1, 0) | -248.23 |
| ASMR | Male | ≥95 years | (1, 1, 1) | -185.63 |
| ASMR | Female | ≥95 years | (1, 1, 0) | -297. |
| ASDR | Both | ≥95 years | (2, 1, 0) | -110.72 |
| ASDR | Male | ≥95 years | (1, 1, 1) | -48.36 |
| ASDR | Female | ≥95 years | (1, 1, 0) | -159.39 |
