# Supplementary material for: Epidemiological transitions of Visceral Leishmaniasis in G20 countries: A 33-year retrospective joinpoint and demographic decomposition analysis with 20-year ARIMA projections
Source: PLoS Negl Trop Dis. 2026 Sep 16;20(9):e0014714. doi: 10.1371/journal.pntd.0014714 (PMC13580953; doi:10.1371/journal.pntd.0014714)
Supplement: Supplementary file 15 [file pntd.0014714.s015.docx]

**S1 Fig** Heatmap of age-standardized rates for visceral leishmaniasis (VL) in G20 countries, 2023. (A) Prevalence; (B) Incidence; (C) Mortality; (D) Disability-adjusted life years (DALYs). Color intensity reflects the magnitude of the rate, with darker shades indicating higher burden. This visualization facilitates direct comparison across countries and indicators.
