# Supplementary material for: Epidemiological transitions of Visceral Leishmaniasis in G20 countries: A 33-year retrospective joinpoint and demographic decomposition analysis with 20-year ARIMA projections
Source: PLoS Negl Trop Dis. 2026 Sep 16;20(9):e0014714. doi: 10.1371/journal.pntd.0014714 (PMC13580953; doi:10.1371/journal.pntd.0014714)
Supplement: Supplementary file 16 [file pntd.0014714.s016.docx]

**S2 Fig** Joinpoint regression analysis of visceral leishmaniasis (VL) age-standardized rates by both sex in Brazil, India, Italy and Turkey aggregate, 1990–2023. (A) Prevalence; (B) Incidence; (C) Mortality; (D) Disability-adjusted life years (DALYs). Solid lines represent fitted trend segments identified by the joinpoint model. The annual percentage change (APC) and its significance (*P < 0.05) are displayed for each segment. The distinct trend phases, including a notable decline post-2011, are clearly delineated.
