# Supplementary material for: Epidemiological transitions of Visceral Leishmaniasis in G20 countries: A 33-year retrospective joinpoint and demographic decomposition analysis with 20-year ARIMA projections
Source: PLoS Negl Trop Dis. 2026 Sep 16;20(9):e0014714. doi: 10.1371/journal.pntd.0014714 (PMC13580953; doi:10.1371/journal.pntd.0014714)
Supplement: Supplementary file 18 [file pntd.0014714.s018.docx]

**S4 Fig** Projected age-standardized rate trends of visceral leishmaniasis (VL) in the ≥95 years age group across the aggregated G20 economies, 2024-2043, based on the ARIMA model. (A–C) Prevalence; (D–F) Incidence; (G–I) Mortality; (J–L) DALYs. Panels A, D, G, and J show the overall trend for both sexes. Panels B, E, H, and K and C, F, I, and L stratify projections for males and females, respectively. Shaded regions represent 95% prediction intervals.
